# Language Origins Viewed in Spontaneous and Interactive Vocal Rates of Human and Bonobo Infants

**DOI:** 10.3389/fpsyg.2019.00729

**Published:** 2019-04-02

**Authors:** D. Kimbrough Oller, Ulrike Griebel, Suneeti Nathani Iyer, Yuna Jhang, Anne S. Warlaumont, Rick Dale, Josep Call

**Affiliations:** ^1^School of Communication Sciences and Disorders, University of Memphis, Memphis, TN, United States; ^2^Institute for Intelligent Systems, University of Memphis, Memphis, TN, United States; ^3^Konrad Lorenz Institute for Evolution and Cognition Research, Klosterneuburg, Austria; ^4^Department of Communication Sciences and Special Education, University of Georgia, Athens, GA, United States; ^5^Department of Speech-Language Pathology and Audiology, Chung Shan Medical University, Taichung, Taiwan; ^6^Department of Communication, University of California, Los Angeles, Los Angeles, CA, United States; ^7^School of Psychology and Neuroscience, University of St. Andrews, St. Andrews, United Kingdom; ^8^Max Planck Institute for Evolutionary Anthropology, Leipzig, Germany

**Keywords:** human evolution, origin of language, bonobo, comparative psychology, infant directed speech, evolution of language, parent–infant interaction, babbling

## Abstract

From the first months of life, human infants produce “protophones,” speech-like, non-cry sounds, presumed absent, or only minimally present in other apes. But there have been no direct quantitative comparisons to support this presumption. In addition, by 2 months, human infants show sustained face-to-face interaction using protophones, a pattern thought also absent or very limited in other apes, but again, without quantitative comparison. Such comparison should provide evidence relevant to determining foundations of language, since substantially flexible vocalization, the inclination to explore vocalization, and the ability to interact socially by means of vocalization are foundations for language. Here we quantitatively compare data on vocalization rates in three captive bonobo (*Pan paniscus*) mother–infant pairs with various sources of data from our laboratories on human infant vocalization. Both humans and bonobos produced distress sounds (cries/screams) and laughter. The bonobo infants also produced sounds that were neither screams nor laughs and that showed acoustic similarities to the human protophones. These protophone-like sounds confirm that bonobo infants share with humans the capacity to produce vocalizations that appear foundational for language. Still, there were dramatic differences between the species in both quantity and function of the protophone and protophone-like sounds. The bonobo protophone-like sounds were far less frequent than the human protophones, and the human protophones were far less likely to be interpreted as complaints and more likely as vocal play. Moreover, we found extensive vocal interaction between human infants and mothers, but *no* vocal interaction in the bonobo mother–infant pairs—while bonobo mothers were physically responsive to their infants, we observed no case of a bonobo mother vocalization directed to her infant. Our cross-species comparison focuses on low- and moderate-arousal circumstances because we reason the roots of language entail vocalization not triggered by excitement, for example, during fighting or intense play. Language appears to be founded in flexible vocalization, used to regulate comfortable social interaction, to share variable affective states at various levels of arousal, and to explore vocalization itself.

## Introduction

### Roots of Language Envisioned Through Cross-Species Comparisons

The claim that language is the quintessential feature of humanity has distant roots (Condillac, 1756). But how did the special power of language begin to arise? Considerable research has been devoted to illuminating roots of the human language capacity through investigation of both gestural (Tomasello and Camaion, 1997; Hopkins and Leavens, 1998; Call and Tomasello, 2007; Call, 2008; Cartmill and Byrne, 2010; Roberts et al., 2012) and vocal communication in other animals (Owings and Morton, 1998; Bermejo and Omedes, 1999; Cheney and Seyfarth, 1999; Nooteboom, 1999; Crockford and Boesch, 2005; Owren et al., 2010; Seyfarth and Cheney, 2010). Research has also addressed notable foundations of cognition in non-humans (Cheney et al., 1986; Bshary et al., 2002; Schusterman et al., 2002, 2003), especially in the great apes (Call and Tomasello, 1994; Povinelli and Eddy, 1996; Herrmann et al., 2007). Such research has revealed much about animal intelligence, including cognitive domains in which animals exceed humans (de Waal, 2016). It has also made clear that even if animals do not display language the way humans do, many of the features of language have foundations in other animals (Snowdon, 2004). Of special interest are the dramatic achievements of a variety of taxa in learning fundamental language features including, importantly, symbolism in the context of human training (e.g., Menzel, 1999; Pepperberg, 2004), especially when it begins early in the life of the animal and is consistent over extended time periods (Griebel et al., 2016). Of particular importance to the present work, there have been notable studies indicating that babbling-like behavior can be observed in species ranging from songbirds to monkeys (Elowson et al., 1998; Bolhuis et al., 2010; Lipkind et al., 2013; Kaplan, 2017; Snowdon, 2018). Research has also provided mounting physiological and genetic evidence about vocalization across species, revealing shared systems as well as species-typical and age-dependent properties of vocal control (DeVoogd et al., 1993; Jürgens, 1995; Wild, 1997; Newman, 2007; Ravbar et al., 2012; Ackermann et al., 2014; Takahashi et al., 2015; Hage et al., 2016; Mello and Clayton, 2016; Roy et al., 2016; Loh et al., 2017).

Still, a gap is assumed, though not quantified—human language is assumed to be vastly more elaborate than any known non-human communication system occurring in the wild. The task we undertake here represents an effort to begin providing quantification of possible fundamental differences in human vocal communication capability as opposed to that of one of our closest relatives, the bonobo (*Pan paniscus*). Our approach is founded in the expectation that maximal comparability may be most evident, and crucial differences may be possible to observe very early in the lives of humans and bonobos.

### Foundations of Language in Human Infant Vocalization

Our research suggests that in the evolution of human language, a key foundational divergence from the primate background may have involved an increasing tendency in the hominin line to vocalize freely, not in the form of language, but in exploratory vocalization with flexible vocal expression of emotional states (Oller, 2000; Oller and Griebel, 2008). In part, this line of reasoning is inspired by the fact that human infants begin life already producing copious exploratory and functionally flexible vocalization (Stark et al., 1975; Koopmans-van Beinum and van der Stelt, 1986; Nathani et al., 2006; Oller et al., 2013; Jhang and Oller, 2017). Such sounds are not language, but the ability and the inclination to produce them flexibly and extensively form crucial foundations for language (Oller, 1981; Koopmans-van Beinum and van der Stelt, 1986; Locke, 1993; Griebel and Oller, 2008).

The early human precursors to speech, termed “protophones” (Oller, 2000), are known to be produced often in the absence of excitement, discomfort, elicitation, or social directivity (Papaeliou et al., 2002; Scheiner et al., 2002; Iyer and Ertmer, 2014), although protophones do also occur in circumstances of high arousal, upset, and celebration. All these patterns are taken as indications of flexibility in protophone control. There is widespread agreement that canonical babbling, which begins in the second half year and includes clearly well-formed, mature-sounding syllables with consonant- and vowel-like components (baba, mama…), is a precursor to speech. Canonical babbling contrasts sharply with cry, laugh, and vegetative sounds such as burps and coughs, which share many features across all primates, and are assumed not to be precursors to language (Stark, 1980; Masataka, 2003).

But the roots of language in human infants appear earlier than canonical babbling. In the first half year of life, protophones such as vowel-like sounds (hereafter “vocants”), squeals, growls, and raspberries, without the well-formed consonant-vowel-like components of canonical babbling, have been argued to form foundations for all subsequent vocal development necessary for language, including canonical babbling ( Oller, 1980; Koopmans-van Beinum and van der Stelt, 1986). These early protophone sounds, like canonical babbling, are typically produced exploratorily and can accompany the full range of positive to neutral to negative affect as reflected in facial expression (Oller et al., 2013; Jhang and Oller, 2017), and although they usually occur at low or moderate arousal, they can also accompany intense emotion and high arousal. It is a requirement of language that all words or sentences be producible at will, at any point that a speaker chooses to produce them, regardless of emotional state or external circumstance. That human infants produce protophones with such flexibility highlights the emergence in very early infancy of a capacity without which further steps in the direction of language are presumably impossible.

### Protophones and Cry

Previously, human infant vocalization was thought to be based on cry, with speech-like vocalization emerging *from* cry (Lester and Boukydis, 1992). Humans were thus thought to begin life with pan-primate vocal capabilities, producing overwhelmingly stereotyped distress signals at high levels of arousal and only diverging from the primate pattern months later. For at least one primate, the common marmoset, the “phee” call does appear to develop *from* infant cry (Takahashi et al., 2015). Yet high arousal, stereotyped sounds are not the only sounds produced by young non-human primates (see e.g., Elowson et al., 1998; Laporte and Zuberbühler, 2011; Snowdon, 2018). Furthermore, cry is not the preponderant vocalization type in *human* infants. On the contrary, *protophones* are the primary vocal expressions of the human infant from the first months. As early as 2–3 months, protophones have been shown to outnumber cry substantially (Nathani et al., 2006; Oller et al., 2013). According to the same studies, by 5–6 months, protophones occur at rates as much as 10 times higher than cries, and earlier work suggests that a preponderance of non-cry sounds holds for at least American, French, and Japanese infants (Bornstein et al., 1992). Furthermore protophones are clearly present from the first day of life (Oller, 1980; Törölä et al., 2012b; Dominguez et al., 2016).

### Endogenous Nature of the Protophones

Whereas cry is uniformly produced by very young infants as an expression of distress and laughter as an expression of playful social connection, protophones express no necessary emotional valence, although they can express distinct and strong emotions on some occasions. Also, protophones are most commonly produced when the infant is not looking toward any potential listener (Oller et al., 2013), suggesting predominant expression of vocal exploration or play, rather than necessary expression of social intentions. More recent data suggest that approximately 70% of infant protophones during naturalistic interactions in the laboratory in the first year are not socially directed as indicated either by gaze direction or by interactive timing (Long and Oller, 2017).

The widely reported claim that infant volubility is enhanced in the context of a vocally active or contingently responsive parent (Rheingold et al., 1959; Weisberg, 1963; Routh, 1969; Bloom and Esposito, 1975) has been disputed, sometimes invoking the opinion that human infants vocalize most when alone (Delack, 1976; Delack and Fowlow, 1978). Given methodological limitations of the cited studies, we are not convinced that infants vocalize most when alone, but are persuaded by this evidence and our own observations that considerable infant vocalization occurs even in the absence of caregiver stimulation.

To view infant vocalization as heavily exploratory, rather than exclusively interactive, is consistent with recent work in modeling of infant vocal development with computer or robotic simulations. These approaches have found success in simulating real infant vocal patterns by treating the modeled infant as a vocal agent with “intrinsic motivation” and vocal “curiosity” (Oudeyer, 2005, 2006; Oudeyer and Kaplan, 2006; Moulin-Frier et al., 2014), implying endogenous vocal exploration. This work is developing general evolutionary and developmental models of vocal communication that balance roles for endogenous exploration and interactive effects ( Warlaumont et al., 2010, 2014; Oller et al., 2016).

### Vocal Interaction in Human Infancy and the Emergence of Language

In spite of the strong evidence for endogenously driven human infant vocalizations, the primary emphasis in prior research has long been on social interaction as the force driving human infant sounds (Anderson et al., 1977; Trevarthen, 1979; Keller and Schőlmerich, 1987; Fernald, 1992; Stoel-Gammon, 1998; Hsu et al., 2001; Gros-Louis et al., 2006, 2014; Goldstein and Schwade, 2008; Menyuk et al., 2014). The emphasis on interactivity is understandable given that content of particular languages must be learned through listening to caregivers and interacting with them. Empirical evidence supports the idea that mother–infant coordination in vocalization is predictive of important linguistic and cognitive outcomes later in life (Jaffe et al., 2001; Hsu and Fogel, 2003; Feldman, 2007).

A potential resolution to the tension between those who see infant protophones primarily as interactive and those who emphasize their spontaneous occurrence is to be found in recognition that natural selection has produced *both* an endogenous human infant tendency to vocalize *and* a strong tendency for infants to attend to (and thus learn to be increasingly responsive to) vocal interaction. Both tendencies have presumably been necessary for the evolution and development of language. Thus, in seeking perspective on the roots of language through comparative research, we need to consider both endogenous and interactive vocal tendencies in humans and related species.

### Absence of Prior Quantitative Comparison of Vocalization Development Across Species

The long-existing claim that humans are far more vocal than other great apes (Morris, 1967; Gardner et al., 1989; Hauser, 1996; Kojima, 2003; Seyfarth and Cheney, 2010) has been taken to be a keystone difference that may have made language a natural aspect of human development but not of other animals. Yet there is a dearth of research quantitatively comparing the most fundamental features of vocal action in humans and other primates. We do not know of a single direct quantitative comparison of rate of vocalization (volubility) across humans and any other great ape; in fact, we know of no volubility comparison across humans and any other *species*. Of particular importance, in our opinion, would be comparative studies of volubility *in infancy* across humans and an ape species, since infants can be presumed to possess relatively high plasticity for learning, a point highlighted in new data along with a review of prior data on vocal learning in non-human primates by Hage et al. (2016). In addition to the lack of infant *volubility* comparison, we do not know of a single direct comparison of the amount of vocal *interaction* occurring within human parent–infant pairs and parent–infant pairs of any other species.

The importance of comparing volubility across humans and other apes, especially of social vocalizations that may reflect a common heritage relevant to the evolution of speech, has been emphasized in theoretical work on the origin of speech in human infants (Locke, 1993, 2009; Oller, 2000; Oller et al., 2016). The key idea is that human infants’ copious protophone production constitutes an activity fostering growth of a flexible vocal capacity and at the same time offering caregivers a basis for vocal interaction that is presumed to foster learning of the ambient language (Elbers, 1997; Stoel-Gammon, 1998; Warlaumont et al., 2014).

### Our Approach to Quantitative Comparison Across Human and Bonobo Infant Vocal Communication

We have chosen to begin by directly comparing data we have been acquiring for the past two decades on vocal communication in human parent–infant pairs with recordings of vocalizations in bonobo parent–infant pairs. The choice of bonobos was partly a matter of accessibility for us to record infants in particular zoos and partly a matter of the fact that bonobos (*Pan paniscus*) are one of our two closest relatives. The optimal cross-species comparators may in fact be bonobos, deemed more vocally active than chimpanzees (*Pan troglodytes*) (Bermejo and Omedes, 1999).

We acquired longitudinal audio-video recorded data on three bonobo infants (3–12 months) and their mothers. The settings for the bonobo recordings were chosen for comparability with other audio-video recorded data available to us on 37 human infants also in the first year of life, data that could be reanalyzed for maximal comparability with the bonobo data. With the bonobo recordings, the goal was to evaluate vocalization in circumstances without significant conflict or fear among the bonobos and in general without high intensity excitement. We thus did not, for example, record during group feeding or during changes of location from one enclosure to another, when groups of bonobos often produce many emotionally charged vocalizations loudly and simultaneously, making it difficult to distinguish individual vocalizers and obscuring any quieter sounds that we expected to be more speech-like. The bonobos were observed in our recordings to be generally peaceful, awake, and alert (we discounted periods when they fell asleep), usually interacting calmly, with grooming, cuddling, and with the infants often being playful. These circumstances were as similar as we were able to observe for recording to those of the human recordings we accessed, circumstances of relatively low or moderate arousal, where human language is particularly salient and thought to be most distinct from other animal communication systems.

One might wonder what role captivity may have played in the emotional states of the bonobos. While they may have experienced stress due to captivity, we were not aware of effects of such stress on the vocalizations or vocal interactions of the bonobos we observed.

We could not and did not try to control the level of arousal of the bonobos, except insofar as we recorded during periods that were expected to show relatively low or moderate arousal. Even so, some excitement, either celebratory or distressful, did sometimes co-occur with vocalizations in both human and bonobo recordings, and when it did, our coding took account of both the vocalizations and their accompanying emotional valence, to the extent that we could judge it. Arousal itself was not, however, a focus of the coding.

Low- or moderate-arousal circumstances are the ones where we presume the roots of language can best be evaluated, because language *requires* the ability to vocalize in the absence of high arousal, although the ability to vocalize in the *presence* of high arousal is also required. For language, humans must be able to vocalize for any purpose and at any time: for social regulation, for sharing of emotions, for social affiliation, for sharing of information about the world, and importantly for no purpose other than to explore vocal sound itself or to play with or practice words or sentences.

Our approach emphasizes the importance of taking observations at the earliest developmental point possible, given that during this very early period, we might be able to document the most fundamental capabilities of the human that make the development of vocal language possible. Moreover, the earliest developing human vocal tendencies would seem to provide a best guess about how ancient hominins first broke away from the ape lineage in terms of vocal capabilities.

The great bulk of research and speculation about the evolution of language has, however, focused on far more elaborate language-like capabilities such as control of joint attention, phoneme production, vocabulary, and syntax (Tomasello, 1996; Deacon, 1997; Kirby, 2001; Christiansen and Kirby, 2003), capabilities that do not appear in the human infant until, at the earliest, the end of the first year of life, or considerably later. Yet infant spontaneous, endogenous vocalization, and caregiver interaction in the first months of infant life appear to form foundations that are crucial for the later developments.

One might ask what forces of natural selection would have favored the capability and inclination to produce protophones in hominin history, given that they occur most often in low- or moderate-arousal circumstances, often announcing no particular social intention or need? Our preferred reasoning (Oller and Griebel, 2005; Griebel and Oller, 2008; Oller et al., 2016) and that of Locke (2006) is based on the fact that hominin infants have long been under selection pressure to produce “fitness signals” because they have long been more altricial (helpless at birth) than the infants in other ape lineages. Due to the longer helpless period in hominins than in their close genetic relatives, there was greater need among hominin infants for caregiver assistance in feeding and protection; thus, hominin infants would have been under selection pressure for capabilities and inclinations that would allow them to advertise their well-being to caregivers (Locke and Bogin, 2006). Since most of life occurs in the absence of high excitement, the production of protophones offered a mechanism for hominin infants to advertise their well-being often, even if caregivers were not looking at them, but were within earshot. Hominin infants whose vocalizations were particularly revealing of well-being were presumably accorded greater caregiver investment, a process referred to by Locke (2006) as “parental selection,” invoking a mechanism suggested by Trivers (1972) and Trivers and Willard (1973). The protophones, according to this reasoning, were thus selected (at least in part) as vocal fitness signals, which had the potential to develop later in life into a panoply of more elaborate and more intentional vocal signals. At each stage of early development, in accord with this reasoning, the protophones would have served multiple functions and would have flexibly expressed a variety of emotional states.

### Research Questions

Regardless of whether parental selection and fitness signaling were the primary mechanisms driving the evolution of the protophones, it seems clear that as we seek vocal phenomena that may reveal foundations of human vocal capabilities in non-human primates, our focus should be directed to flexibly used sounds. Thus, we sought to record and code sounds that can be produced by non-human primates in a variety of circumstances, especially without high emotional intensity. Of lesser interest would be vocalizations showing stereotyped association with particular states of emotion, as for example, cries/screams, which are associated with negative or laughter with positive states.

Judgments of functions and affect of communications in non-humans are no trivial task, and we acknowledge that it is only possible to compare the functional usage of vocalizations across humans and other species (perhaps especially in infancy) at a fairly global level. However, the task of such comparison is important, and we will present a method below that has yielded preliminary insights about functional use of vocalization in bonobo infants that will hopefully be expanded and improved in the near future.

Our approach also emphasizes comparisons of vocal categories collapsed to provide three similar groupings across the infants of both species. We chose this low granularity for comparison because it provides a conservative framework, discriminating between the most emotionally negative (human cry/bonobo scream) and positive (human and bonobo laughter) vocalizations that occurred in the samples for both species and leaving all other non-vegetative vocalizations to be treated as potential speech-like material in both cases. In the human case, this third category encompasses the protophones, for which there is considerable empirical and theoretical reason to view them as speech precursors. In the bonobo case, the questions about potentially protophone-like material are empirical, leading to two study questions about the infant vocal types:

(1) How frequently did protophone or protophone-like vocalizations occur in both species?
(2) To what extent did bonobo infants produce protophone-like vocalizations in functionally similar ways to human infants’ protophones, in particular showing similar patterns of emotional/affective valence and/or playfulness?

Two additional questions concerned vocal interaction between parents and infants in the two species:

(1) To what extent did both human and bonobo parents communicate vocally with their babies?
(2) To what extent did human and bonobo parents and infants engage in vocal turn-taking?

## Materials and Methods

### Research Participants

#### Bonobos

We recorded the mother-infant pairs longitudinally during the first year of the infants’ lives: in the Memphis Zoo, Kiri (20 years old) with Mobali (4 months old at the beginning of the recordings and 11 months at the end), and from the Leipzig Zoo, Yasa (16 years old) with Kasai (6–12 months) and Lexi (14 years old) with Yaro (3–7 months). All three infants were male. Aside from an occasional cold, all the animals present in any recordings were healthy.

The recordings of the bonobos were made from outside the spaces that the animals regularly visited and included no human physical contact with the animals. They were made with permission from the Leipzig and Memphis Zoo administrations. Both zoos follow WAZA regulations for animal care.

#### Human Subjects for Comparisons

The recordings of humans for the three comparison studies included a total of 37 infants. Recordings were made with written informed consent of all the parents and under approval from the Institutional Review Board of the University of Memphis or the University of Georgia. The human infants were recruited with their parents as part of three different studies for longitudinal research to begin as near birth as possible and to continue across at least the first 2 years of life. There were no reported risk factors for developmental disabilities or hearing impairments. All the families were of low-middle to high socioeconomic status (SES). Most of the infants heard only English at home, but a few heard other languages as well. There were, however, no notable differences discerned in volubility of infants or in interactive patterns based on ambient languages at home or SES, and so all the data are presented without differentiation regarding ambient language at home or SES.

The first work (Oller et al., 2013) from which we drew comparison data, hereafter the “Memphis1 study,” provides detailed demographic information about the nine human participants (seven female) all recruited from the area near Memphis, TN, United States, for laboratory audio–video recordings. The Memphis1 data included coding of the vocal categories deemed optimal for comparison with bonobos (cry, laughter, and protophones), as well as coding of various features of interaction requiring good video views, thus offering a basis to assess vocal functions and interaction. The Memphis1 data were reanalyzed and restructured for the comparisons presented here.

The second study (Iyer et al., 2016), hereafter the “Athens study,” provides demographics for 16 infants (seven female) recruited from the Athens, GA area, also for laboratory recordings with audio-video. The Athens study assessed human infant protophones in three recording circumstances to offer perspective on different interactional effects in human infant vocalizations. The data were restructured for the comparisons presented here.

No data from the third work, hereafter the “Memphis2 study,” have been previously published in print. The effort included all-day audio-only recordings made with LENA recorders (Zimmerman et al., 2009) and obtained longitudinally in the homes of 12 (six female) infants from the Memphis area. The Memphis2 study offers data on randomly selected recording segments from the maximally representative circumstance of the infants’ homes.

For additional methods descriptions see Supplementary Material, in the file labeled Supplementary Table S1.

## Results

### Acoustic Overview of the Infant Vocalizations of Both Species

**FIGURE 1:**
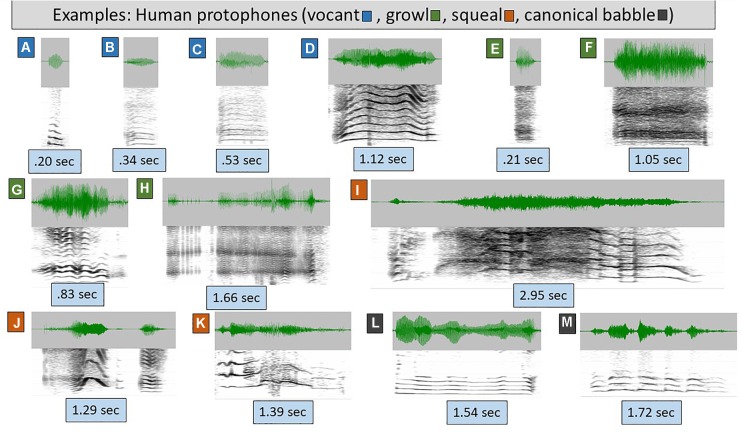
Human protophones. The spectrograms (range 4–5 kHz, 30 Hz bandwidth) and waveforms illustrate human protophones, which come in extremely variable form, as illustrated. Even these examples vastly underplay the acoustic variability of protophones. **(A–D)** are categorized as vocants, the most prototypical human infant vocal type, with consistent harmonic spacing and little or no dysphonation (indicating modal voice, the overwhelmingly typical human phonatory pattern in speech). The sounds were produced by 1–3 month-old typically developing human infants. Vocants are often as short as 0.1 s but can be as long as 3 s. Their intonation is not always smooth, but may involve notable variations as in **D**, where the rise and fall of the harmonics across time signals intonational variation. **(E–H)** are growls, also from 1–3 month-olds. In growls, phonation is harsh (i.e., it is chaotic, and harmonics are absent or less prominent than in vocants) as in **E** and **F** or creaky (consisting of a pulse regime, including prominent spikes in the waveform) as in **G** and **H**. As with vocants, growls can be very short or very long. **(I–K)** are squeals, from 0–3 month-olds. Squeals always show very high pitch (*f_0_*) as seen in widely spaced harmonics during at least a significant portion of the utterance. As with vocants and growls, squeals can be very short or very long, and as with vocants, they can involve considerable intonational variation, as seen in all three presented examples. **(L,M)** are reduplicated canonical babbles from 11 month-old infants. This is a vocal type that has never been documented to occur in any non-human primate even with human training. Canonical babbling involves rhythmic modulation of the acoustic waveform by movements of the jaw, lips, and/or tongue during modal phonation. From a phonatory standpoint, canonical babbles are vocants, but their supraglottal articulations result in a special pattern of well-formed syllables, adaptable for speech.

**FIGURE 2:**
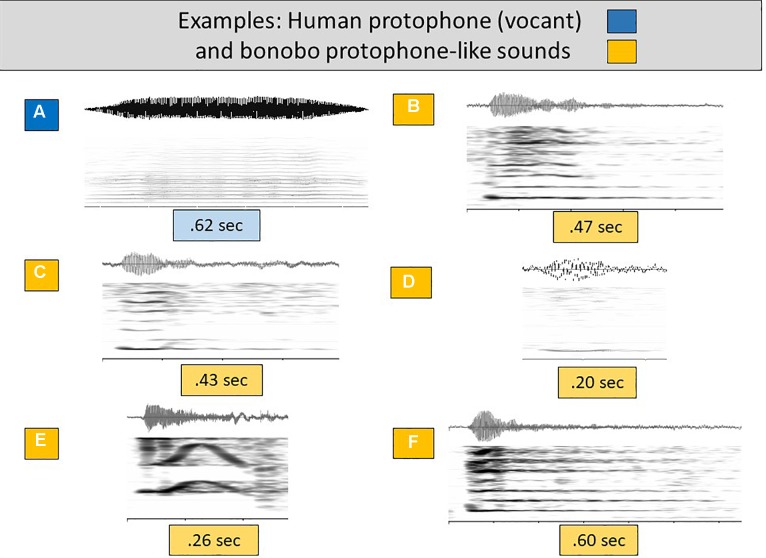
Human and bonobo sounds of the speech-like grouping. **(A)** offers a spectrogram and waveform to illustrate an additional human (3 months) vocant, selected as a particularly prototypical human infant protophone, with consistent harmonic spacing and a smooth intonational pattern involving little or no dysphonation. **(B–F)** are similarly composed displays showing bonobo infant sounds deemed auditorily similar to (that is, pertaining to the acoustic range encompassed by) human protophones, all including laryngeal phonation and clear harmonic energies. These bonobo sounds appear to be acoustically similar enough to the most common human protophones (vocants, squeals, and growls) that we treat them as candidates for speech-like material.

**FIGURE 3:**
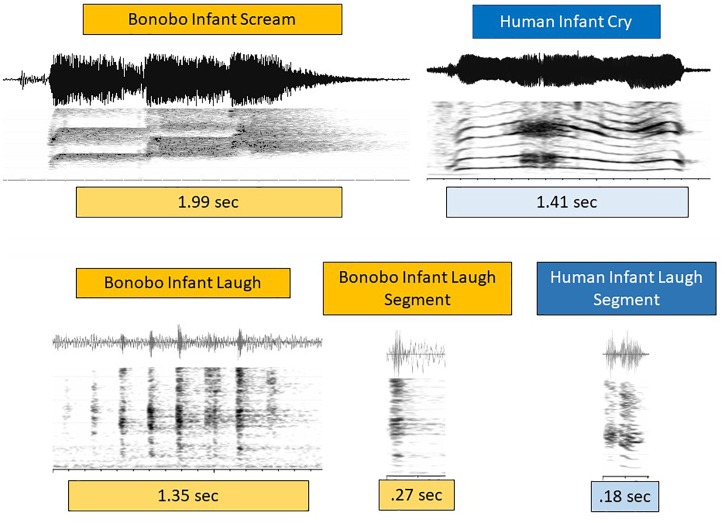
Cries/screams and laughs in human and bonobo infants. In the first row, a spectrographic and waveform display of an infant bonobo scream in three bursts at high pitch (*f_0_*), each burst about 600 ms, is contrasted with a prototypical but acoustically quite distinct pattern of human infant cry. Prototypical human infant cry often occurs as a continuous phonatory event, including at least one period of distinct dysphonation, as seen in the spectrogram beginning at about 500 ms. These bonobo and human negative vocalizations are thus similar in typically showing notable dysphonation, but very different in the timing of its occurrence. They are also different in that human cry, while it can occur at high *f_0_* (roughly pitch), is typically produced at much lower *f_0_* than the bonobo screams we observed. Based on the functional similarity of infant bonobo scream and human infant cry, we treat them as analogous in spite of acoustic differences. The second row displays a multi-segment infant bonobo laugh, followed by a single bonobo infant laugh segment, compared with a human infant laugh segment. The laughs differ from scream/cry in that their bursts and nuclei tend to be much shorter in both species. Laughs differ across the species in that bonobo laugh often consisted of an ingressive-egressive pattern rather than a sequence of egressive bursts (as in the figure), while human infant laughs are overwhelmingly egressive, consisting of a glottal burst (as in the figure) followed by a brief voiced nucleus. Again functional similarly of the sounds called laughter in the two species (both occurring as playful, joyful expressions) leads us to treat them as analogous in spite of their acoustic differences.

To orient the reader to the types of sounds occurring in the data, Figure 1–3 provide waveforms and spectrographic examples from human and bonobo infants (the 24 corresponding sound files can be found in Supplementary Material, in the file labeled Supplementary Data Sheet S1). The human infant vocalizations in all the studies were coded for vocal type in such a way that they could be collapsed to the three groupings we settled on for comparison with the bonobo infant vocalizations. Human cry was treated as analogous to bonobo scream, human and bonobo laughter were deemed analogous, and all the human protophones (squeal, growl, vocant, raspberry, whisper, ingress, etc.) were treated as analogous to the “other” category of potentially protophone-like sounds of the bonobo infants.

Figure 1 illustrates the extreme variety of the most common human protophones of the first 6 months of life, vocants (Figure 1A–D), growls (Figure 1E–H), and squeals (Figure 1I–K). The remaining two displays (Figure 1L,M) represent reduplicated canonical babbles of human infants, a type of vocalization that tends to occur only after 6 months of age and resembles mature speech substantially. The well-formed syllable structure of reduplicated canonical babbling has never been observed in any non-human primate to our knowledge.

Figure 2A presents a particularly prototypical human infant protophone of the first 6 months of life, a vocant, for comparison with protophone-like infant bonobo sounds (Figure 2B–F). The vocant was generated by laryngeal phonation, with evenly spaced harmonics and a smooth intonation pattern. Like the human protophones, the infant bonobo sounds were also primarily generated by laryngeal phonation, with evenly spaced harmonics, providing justification for treating them as potential speech-like material. Figure 2E presents an example that can be analogized to a human infant squeal (see Figure 1I,J,K and accompanying wave files). Figure 2B,C,F can be analogized to vowel-like sounds although they are auditorily more aperiodic than most human vocants and have sharper onsets (revealed spectrographically in darker harmonics near the onset—see for comparison Figure 2A and 1A–D, and accompanying wave files). Figure 2D also looks spectrographically like a human vocant, although again the vocalization sounds more aperiodic than most human vocants.

Figure 3 presents spectrographic and waveform displays of infant bonobo scream and human infant cry on the first row, and bonobo and human infant laugh on the second. Bonobo infant scream was typically composed of one or more roughly half-second bursts of shrill (very high *f_0_*) and dysphonated sound, as illustrated. The prototypical human cry in Figure 3 consisted of a fairly continuous phonatory event at much lower *f_0_*. Dysphonation occurred in both cases, but the timing patterns and spectral concentrations were very different. Still, all the examples in the data of bonobo scream and human cry were interpreted as expressing a high degree of negativity. The functional similarly between infant bonobo scream and human infant cry justifies treating them as closely related (de Waal, 1982).

In the second row of Figure 3, laughter events again illustrate both similarities and differences in the acoustic patterns across the species. Short duration bursts were the hallmark of laughter for both species, but human infant laughs were more commonly based on egressive airstream throughout, with each unit consisting of a glottal burst followed by a brief phonated nucleus. Bonobo infant laughs required no burst-nucleus sequence, and could consist either of a series of egressive glottal bursts or of an audible sequence of rapidly alternating ingresses and egresses either with or without phonation, a pattern that we have never observed in human infant laughter. In spite of these acoustic differences, the laughter in both cases functioned similarly in, for example, physical play and tickling, and we resolved as others have done (Davila Ross et al., 2009, 2010), to treat them as closely related.

### Rate of Production of Each of the Three Vocal Types in Seconds per Minute Across the Infants of Both Species

**FIGURE 4:**
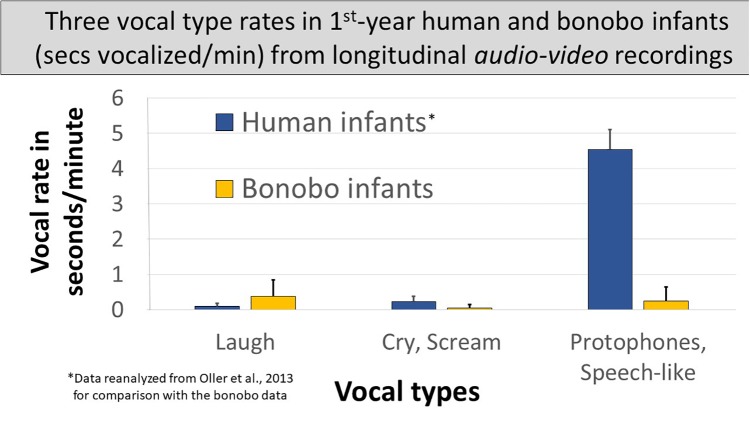
Three vocal types of human and bonobo infants in seconds vocalized per minute. The figure displays vocal seconds/minute in human and bonobo infants in the first year by vocal type, human data derived from audio–video recordings of the Memphis1 study. Individual bonobo and human infant laugh rates overlapped. Distributions also overlapped for cry/scream. Protophones in humans, the sounds regarded as precursors to speech, occurred far more frequently than any other vocal type from either species, and individual human protophone rates did not overlap with rates for any other vocal type for either species; 95% confidence intervals are displayed.

Spectrographic inspection suggested utterance durations differed between the species and across the three vocal types. So we first compared vocalization time (seconds vocalized per minute) for each vocal type across the species. Figure 4 compares seconds/minute across all the non-vegetative sounds produced by either species, merged into the three vocal types. In the bonobo infants: (1) laughs accounted for > ½ the vocalization time across the three vocal types^1^ at a rate of <0.4 s/min; (2) screams occupied less time, <0.1 s/min, than laughs^1^; and (3) the most protophone-like bonobo sounds occurred at an intermediate rate between bonobo laughs and screams, 0.24 s/min. The human data in Figure 4 are based on reanalyzed data from the Memphis1 study: (1) laughs occupied only a small portion of human vocalization time and less than in the bonobo infants^1^; (2) human infant cries showed considerably higher seconds/minute values than bonobo infant screams^1^, but still represented only a small proportion of the human vocalizations; and (3) human infant protophones showed by far the highest seconds/minute values (>4.5 s/min) of any vocal type for either species, 14 times higher than human laughs and cries combined, 6.5 times higher than all three infant bonobo vocalization types combined, and more than 18 times higher than the most speech-like infant bonobo sounds. The lowest of the nine individual human protophone rates was nearly six times higher than the highest of the three infant bonobos’ rates of protophone-like vocalizations.

The function/affect judgments during infant vocalizations were more workable for humans than for bonobos (see Supplementary Material for details on coder agreement). Acknowledging this limitation, we offer the following tentative observations about function/affect accompanying vocalizations in the two species. Human protophones were relatively *rarely* (<14% of the time) coded as having negative emotional valence based on facial affect, while the most protophone-like infant bonobo sounds were *typically* (>60% of the time) coded as negative (i.e., as complaints or pleas), and the remaining protophone-like infant bonobo sounds were coded overwhelmingly as “don’t know,” meaning the coder was unable to assess the emotional valence of the event, often because the infant or other interactive participant was not visible on the video during the event. Perhaps most importantly, the infant bonobos were never observed to produce vocal play or vocal exploration, a pattern that was commonly observed in the human infants and has been observed in much prior research (Stark, 1980; Papoušek, 1994; Jhang and Oller, 2017). The commonness of vocal play in human infants will be addressed empirically under the Memphis2 study, below.

### Rate of Protophone and Protophone-Like Sounds in Seconds per Minute at Two Points in the First Year

**FIGURE 5:**
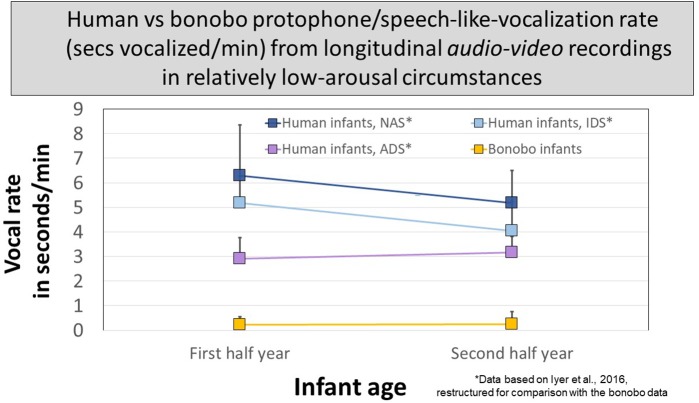
Protophone rates for human infants compared with rates of candidate speech-like sounds produced by bonobo infants. The most speech-like infant bonobo vocalizations in seconds/minute from audio–video recordings of early and late in the first year of life occurred far less than protophones from human infants similarly recorded in the Athens study. The Athens data are broken down for human caregivers (1) present but silent (NAS, no adult speech), (2) present and speaking to infants (IDS, infant-directed speech), or (3) present but speaking to another adult (ADS, adult-directed speech). In all three circumstances, the human protophone rates were dramatically higher than those of the bonobo infants; 95% confidence intervals are displayed.

Figure 5 presents data for human protophones from the Athens study restructured for comparison with the protophone-like bonobo sounds at two ages. The human data are segregated to reflect circumstances, as per instructions to mothers during recordings: (1) no adult speech (NAS), (2) infant-directed speech (IDS), or (3) adult-directed speech (ADS, between the caregiver and another adult). Human infant protophones occupied ∼3 s/min with ADS and occupied significantly more time with NAS and IDS, >4 s/min. There were no statistically significant differences in protophone rate between the IDS and NAS circumstances. The ample production of protophones by infants not in interaction (NAS) suggests a deep human endowment for endogenous vocalization, requiring neither distress, nor high arousal, nor social stimulation—it was typical for human infant protophones observed in the studies to show no distress, no high arousal, and to be produced in the absence of social interaction.

The bonobo mothers never vocalized to their infants, so protophone-like bonobo sounds are pooled for comparison with the human infants in the three circumstances in Figure 5. In all circumstances at both ages, human protophones showed >11 times the seconds/minute rate of the bonobos’ most protophone-like sounds (<0.3 s/min).

### Vocalization Rates in Utterances per Minute for Human Infants and Mothers Vocalizing to Infants Across the First Year in Laboratories and in Randomly Selected Samples at Home

**FIGURE 6:**
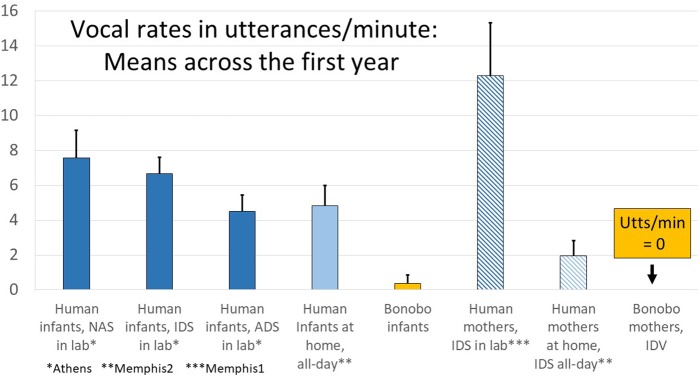
Laboratory and all-day at home human infant vocalization rates along with infant-directed speech/vocalization (IDS/IDV) rates in humans and bonobos. The data provide comparisons from all the human studies (Memphis1, Athens, Memphis2) displayed in utterances/minute. As indicated on the right of the display, while human parents produced considerable IDS (>12 utterances/min in the laboratory and almost 2 utterances/min in randomly sampled segments from all-day recordings), bonobo mothers produced no IDV at all in the recordings. The human parents produced far more IDS in the laboratory than at home, and in the laboratory, they produced about twice as many IDS utterances/minute as human infants produced protophones, a pattern that appears to correspond to a parental “teaching” mode or perhaps a style adopted for the camera. At home, the patterns were very different, with parents producing far less IDS. In fact when the human infants were awake in randomly sampled segments from all-day recordings, the laboratory pattern was reversed, and the infants produced more than twice as many protophones as their mothers produced IDS utterances. Further the rate of infant vocalizations in randomly selected samples at home was about the same as the rate occurring during adult-directed speech (ADS) in the laboratory.

In Figure 6, the data are presented in utterances/minute to afford comparison of data from the all-day recordings of human infants in their homes (Memphis2), where the data were based on real-time coding (RTC, which is necessarily utterance-based), with data from the Memphis1, Athens, and bonobo studies. Human parents in the laboratory produced >12 utterances/minute of IDS (from Memphis1), but only about 1/6 that much in all-day randomly sampled segments when the infant was awake (from Memphis2). Still, two utterances of IDS/minute represents a considerable amount of talk to the baby—120 utterances per hour. As noted above and represented in Figure 6, the bonobo mothers did not produce any infant-directed vocalization (IDV) at all.

Figure 6 also illustrates that human infants produced considerable numbers of protophones (4.8/min when they were awake), even in their home environments with random sampling (Memphis2). While parents tended to produce much more IDS in the laboratory than at home, human babies manifested high vocalization rates in both settings. The endogenous tendency of human infants to produce protophones is reflected especially in the fact that, when human infants were deemed to be alone and awake according to the Memphis2 study questionnaire (there were 220 five-min segments where infants were coded as alone and awake), they produced an average of 4.0 protophones/min, a quantity suggesting practice-like exploration. One might speculate that the infants alone were vocally active not due to an inclination to explore vocalization but by upset at being alone. However, 60% of the alone segments in the Memphis2 data included no crying or whimpering, and 93% included at least some protophones, with 74% including at least 1 protophone/min. Throughout all the human data, protophones vastly outnumbered cries and whimpers, and even in the alone circumstance at home, human infants produced 6.7 times more protophones than the sum of cries and whimpers.

While human infants produced protophones often for their own exploratory purposes, they seemed to be quite responsive to IDS in the laboratory as indicated by the subsample coded for illocutionary force from the Memphis1 study. The subsample data showed that during the designated interactive sessions when mothers produced >12 IDS utterances/min (Figure 6), infants produced protophones at >5/min and that more than half their protophones during those sessions were interpreted by the coders as directed toward the parent.

In the Memphis2 data, the human infants gave additional indications of their endogenous vocalization tendency. In these randomly sampled segments from all-day recordings, they produced more than twice as many protophones as parents produced IDS utterances. Furthermore, in response to the question about whether infants engaged in pure vocal play or vocal exploration, the coders indicated that 89% of segments where the infant was awake included vocal play with protophones. At the same time, the infants did not ignore parental entreaties to conversation even in the home recordings, where parents spoke to them at lower rates than in the laboratory. In fact, if a parent talked to an infant at all in a 5-min segment at home (and they did so in 69% of segments where the infant was awake according to responses on the question answered by coders’ about IDS), the infant responded with at least some vocal turn taking in 22% of the segments according to the coders’ questionnaire responses. If the parent was more vocally insistent, attempting to engage the infant in conversation more often (as suggested by a high rating on the IDS question, indicating IDS occurred at least half the time), the infant responded with vocal turn taking in more than 2/3 of the segments.^2^

## Discussion

### Provisos

The work presented here is the first direct quantitative comparison of vocalization rate and vocalization interaction rate between human infants and infants of any other species. It is, however, a first step. As such, we acknowledge a number of limitations.

Although we have compiled considerable data on human infant vocalizations for comparison here, the amount available to us on bonobo infants was much smaller and more circumstantially limited: our coded observations were made on only three infants and their mothers in captivity, and the longitudinal sampling was not as well-spaced across the year as in the human studies, which included 37 infants. It will be important for future efforts to augment the data to make them more comparable in size and sampling. In addition, expanding the circumstances of observation is critical, especially with observation of non-human infants in the wild. It is hoped that a substantial number of bonobo and other primate infants can be observed systematically in circumstances that appear to stimulate vocalization, including play and other social interaction with conspecifics of various ages and genders. We hope also to see improved methods for making judgments about function/affect in the non-human infants during vocalization. Our methods and dataset should provide a foundational step, facilitating such comparative communication research in the future.

### Summary of Outcomes

#### Empirical Support for Language Foundations in Bonobos

Our results offer support for prior suggestions about foundations of human language in non-human primates. Bonobo infants produced a considerable number of vocalizations in low- to moderate-arousal social circumstances (>600 of them, about one every 3 min), and there is reason to analogize those sounds to the protophones of human infancy partly because they occurred predominantly in low- to moderate-arousal social circumstances. Prior research in operant vocal learning paradigms suggests flexibility in vocal production of primates is relatively low in adulthood (see review in Seyfarth and Cheney, 2010), but more flexible in infancy (see review in Hage et al., 2016), and we presume that the low- to moderate-arousal sounds of the bonobo infants observed in the present work represent the sort of vocal material that can be enhanced and modified with experience.

#### Much Greater Speech-Like Vocal Activity in Human Than Bonobo Infants

The potential for vocal modification through experience would, however, appear to be vastly greater in human infants. Even in all-day home recordings, they produced speech-like vocalizations at rates > 12 times higher than the bonobo infants, and there was no overlap between speech-like vocalization rates for any of the human infants examined here (either in the laboratory or at home) and any of the three bonobo infants in the study. The result suggests an evolved tendency in the human infant to produce copious vocal raw material that can be brought to the service of flexible communication (but see comments below on variations in volubility of infants and IDS across human cultures and conditions of SES).

#### The Tendency for Exploratory Vocalization in Human Infants

The human infants we studied, often produced speech-like sounds, the protophones, when they were alone and apparently comfortable, suggesting a strong endogenous tendency to vocalize playfully or exploratorily. Physical play was of course common in the bonobo infants, but there was not a single vocalization other than laughter produced by any of the bonobo infants that was deemed to be motivated by playfulness based on the utterance-by-utterance coding of activities of the infant and any interactor. Laughter seemed always to be a response to external stimulation (often tickling or rough and tumble play) in the bonobo infants. In fact, for all three vocal groupings (scream, laugh, and protophone-like sounds), the bonobo infant vocalizations were overwhelmingly interpreted as responses to (1) external threats or irritations, (2) physical discomforts, (3) frustration, or (4) joyful experience of interactive physical play (laugh only). There may well have been bonobo infant vocalizations in the sample that were generated endogenously by infant interest in the sounds themselves, but no vocalizations were observed to constitute such vocal exploration. In contrast, nearly nine-tenths of the 5-min recording segments randomly selected from all-day recordings where the human infant was deemed awake included, according to the coders, at least some pure vocal play or exploration.

#### Massive Differences in Vocal Interactivity Across the Species

Infant-directed speech and vocal interaction are viewed as critical in language development (Brazelton et al., 1974; Stern, 1974; Jaffe et al., 2001; Goldstein and Schwade, 2009). Vocal interaction comparisons across the species revealed even more dramatic contrast than in the case of infant vocalization rates. The human parents produced IDS copiously, both in the laboratory when they were expected to interact for recordings and in all-day recordings at home, randomly sampled, where no such expectation existed. Yet, although all the bonobo mothers vocalized toward other bonobos (63 total instances were coded, often seemingly directed toward bonobos that could be heard vocalizing loudly outside the enclosures where the recordings occurred), we observed not a single case of a bonobo mother vocalizing toward her infant, nor of any other bonobo vocalizing toward an infant. This is not to say the bonobo mothers were unresponsive. On the contrary, they responded quickly and comfortingly to their infants’ complaints and pleas, for example, by looking toward them, picking them up, or nursing them. It is also worthy of note that the number of bonobo mother vocalizations observed was only about one-tenth as many as the number of bonobo infant vocalizations observed.

### Additional Quantitative Perspectives on Vocalization in Our Closest Primate Relatives From Prior Research

We can construct an additional potentially useful comparison here based on the sparse prior literature about chimpanzee (*Pan troglodytes*) vocal development. A female chimpanzee called Pan was raised from birth by humans. The Japanese researchers talked with Pan, as with a human infant, reinforcing and counting her vocalizations 10 h daily from birth through her 18th week (Kojima, 2003). Pan’s highest volubility from 8–18 weeks was 0.14 non-distress utterances/min, all portrayed as “grunts,” which occurred during face-to-face interactions with human caretakers. While this rate was less than half that observed here for bonobo infant protophone-like sounds, the researchers reported it to be notably higher than grunt rates occurring in two other chimpanzee infants who were not given the human interaction experience.

In another study, a European group (Laporte and Zuberbühler, 2011) conducted >450 h of observation of 14 young chimpanzees in the wild, reporting grunt rates lower than Pan’s in the same age range, though there is reason for concern that many grunts in this study conducted in the wild may not have been audible to the observers and that there could have been additional potentially protophone-like vocal types that were not coded in either of the chimpanzee studies. The Japanese researchers attributed the relatively high grunt rate produced by Pan to consistent human vocal elicitation, although it was also the case that Pan was given milk as a reward for vocalizing (Kojima, 2003).

As far as we know, the only case quantified thus far of maternal utterances directed to a chimpanzee (or bonobo) infant involved the human-reared Pan, who later raised her own daughter Pal, occasionally vocalizing *to* her. The researchers observed no vocal interactions in two other chimpanzee mother–infant pairs at ∼24 months, but Pan/Pal showed ∼0.1 vocal turns/min at that age (Kojima, 2003). Also, during the first 18 months of the infants’ lives, the three mother–infant pairs were observed during regular 10-min periods. Only Pan/Pal showed consistent vocal interaction across the samples. The rate of vocal interaction was lower than in the 24-month sample (maximum ∼0.02/min). Pan vocalized to Pal, through 18 months, including cases without a vocal response from Pal, at ∼0.05/min.

Of course even Pan’s rates of IDV up through 18 months were low compared with human rates, ∼40 times lower than the rate suggested by the current Memphis2 study for human infants recorded all day at home in the first year. But even so, the Japanese report provides an intriguing suggestion that cross-generational stimulation can enhance vocal interactivity in our close phylogenetic relatives.

In states of high arousal, infant vocalization rates may be higher than those reported here for both humans and other apes. It has been claimed that mixed-age bonobo groups in the wild vocalize more than the usually smaller captive groups, and that mother–infant vocal interaction *does occur* in bonobos (Bermejo and Omedes, 1999). Furthermore, some monkeys appear to be much more vocal in general than either chimpanzees or bonobos (Owren et al., 1992; Takahashi et al., 2013), although, again, quantified cross-species comparisons are absent. Eventually, it should be possible to compare vocal rates of mature non-humans to the mature human speech rate which has been quantified from randomly sampled naturalistic recordings at ∼16 words/min (4–6 s/minute of vocalization) every waking hour (Mehl et al., 2007).

### Cross-Cultural and Socioeconomic Perspectives on Human Infant Vocal and Vocal Interaction Rates and the Apparent Robustness of Human Vocal Learning

Our observations should be tempered by reported cultural and SES differences in amount of IDS used by humans (Lieven, 1994). While many cultures/languages show notable IDS rates (Fernald et al., 1989), a substantial number of reports have claimed that cultures exist where very little if any IDS occurs (Dixon et al., 1981; Ochs and Schieffelin, 1984; de León, 1998; Rabain-Jamin, 2001; LeVine, 2004; Correa-Chávez and Rogoff, 2009). Furthermore, there is widespread evidence that IDS differs across SES (Hart and Risley, 1995; Hoff et al., 2002; Hoff, 2003; Hirsh-Pasek et al., 2015), presumably another indication of strong cultural differences regarding caregiver inclination to engage infants vocally. Even in the current research, with SES ranging relatively narrowly, we found dramatic differences in rates of IDS across individual families. While all parents were observed in our Memphis2 study to produce IDS sometimes in the home sampling, the amount of IDS showed a 15-fold difference across the 12 families (mean = 1.9 utts/min, maximum = 4.8, minimum = 0.32).

Quantification of purported low rates of IDS in some cultures has been limited. Studies that have offered the most detailed information we know of do not suggest that caregivers in the low-IDS societies produce *no* IDS, nor that infants in these societies produce *no* protophones, but rather that interaction rates are lower than in more high technology societies, and compared, for example, with non-vocal interaction rates (Mesman et al., 2018). It has also been noted that prior emphasis on low interaction rates of parents with infants may have distorted the picture because other caregivers, such as siblings, that may play important roles in interaction, have been not been taken into account (Whaley et al., 2002).

A recent new body of evidence on rates of IDS in the first year of life has been presented by Cristia et al. (2017) regarding a “pre-industrial” culture in lowland Bolivia (the Tsimane), and including a review of additional older data on IDS from preindustrial cultures of Guatemala (Klein et al., 1977) and southern Africa (Konner, 1977), along with both low and mid SES families in Boston (Tulkin and Kagan, 1972). The reports indicate that IDS is present, not absent in any of the cultures. The Tsimane of Bolivia showed the lowest rate of IDS (about 42 s/h). The !Kung of southern Africa showed a rate several times higher than the Tsimane, the same rate as for the low SES families in Boston (360 s/h). The mid SES families in Boston were reported to produce the highest rate, 600 s of IDS per hour.

The studies reviewed by Cristia et al. (2017) were reported in a way that indicated neither numbers nor durations of IDS utterances, but rather assessed numbers of fixed intervals per unit time during which IDS occurred. Thus, we cannot unambiguously compare the rates observed for IDS in Memphis2 with those reviewed by Cristia et al. (2017). A loose comparison can, however, be constructed if we assume that IDS utterances in our study had average durations of about one and a quarter seconds per utterance, an estimate based on spectrographic measurements of IDS utterances in our sample. Converting our observed rates on this basis to hours per minute, the Tsimane rate of IDS (42 s/h) was lower than the mean of the 12 families in Memphis2 (143 s/h), but higher than the lowest rate observed among the 12 families (24 s/h). A more trustworthy comparison would require utterance counts and spectrographic measurements of individual utterance durations across the cultures compared, and we hope to have the opportunity to conduct such a study in the near future.

If we wish to place our finding of bonobo lack of IDS (for just three captive pairs) in the perspective of the full range of variation in human cultures, the most useful sources of estimates should, we contend, be obtained from random sampling of all-day home recordings. Given that the human parents we observed produced six times more IDS in the relatively brief laboratory recordings than during randomly selected segments from the home (where parents were not being observed by research personnel), we suspect prior estimates of amount of IDS (as for example those reviewed by Cristia et al., 2017) may have been inflated because they tended to rely on time periods during which parents knew they were being observed. Note that the Memphis2 mean IDS value estimated from randomly selected samples at home and converted by the method described above was considerably lower than the value attributed to either low or mid SES families in Boston (143 s/h for Memphis2 vs 360 for low SES and 600 for mid-SES in the Boston families).

Note further that even the family in Memphis2 that produced the lowest rate of IDS, the lowest rate we know to have yet been reported for any human family, produced well over 200 IDS utterances per 12-h day. If the bonobo mothers had produced IDV at the same rate, we would have found not fewer than 500 cases of bonobo IDV. Of course, even our lowest rate is surely not the actual minimum for humans. It is uncertain how low the rate can be without significant negative effect on infant speech learning. There are numerous studies suggesting that IDS is important for infant speech and cognitive development and that higher SES corresponds to higher rates of IDS (e.g., Hart and Risley, 1995; Hoff et al., 2002; Hoff, 2003; Hirsh-Pasek et al., 2015; Schwab and Lew-Williams, 2016; Gilkerson et al., 2017, 2018).

Differences in infant *volubility* have also been argued to be important in language development (Eilers et al., 1993; Oller et al., 1995), a claim that was not confirmed in a long-term follow-up study based on the all-day LENA recording and automated analysis method (Gilkerson et al., 2018). The human propensity to acquire language appears to be robust. Even in the first year of life, the most salient single event in vocal development, canonical babbling (dada, nana…), shows an onset that is remarkably similar (5–8 months) across major variations in SES, premature birth, and ambient language (see review in Oller, 2000). The conditions that have been reported to retard the onset of canonical babbling are, to our knowledge, all associated with clinical disorders of communication: hearing impairment (Oller and Eilers, 1988; Vinter, 1994), Down syndrome (Lynch et al., 1995), William syndrome (Masataka, 2001), cleft palate (Chapman et al., 2001), autism (Patten et al., 2014), and fragile-X syndrome (Belardi et al., 2017).

It has been reported that infants of low SES show lower volubility (overall protophone rate, undifferentiated with regard to vocal types) in laboratory recordings than infants of higher SES (Eilers et al., 1993; Oller et al., 1994, 1995). Also blind infants have been reported to show low volubility (Fraiberg, 1979). On the other hand, additional factors that are known to constitute or have been thought to potentially constitute risk factors have *not* been found to correspond to low volubility. Perhaps the most salient of these is hearing impairment (including congenital profound deafness), which has been claimed, without clear empirical support, to yield low volubility (Lenneberg, 1967). In fact, a variety of empirical opportunities to confirm this suspicion have failed to yield any such evidence (Koopmans-van Beinum et al., 1998; Nathani et al., 1999, 2000, 2007; Clement, 2004). Also infants growing up with two languages have not exhibited low volubility (Oller et al., 1997).

Longitudinal research across the first year has found infants born prematurely without additional significant perinatal risks (and ∼1500 g birthweight) to show volubility similar to that of full-term infants (Beckwith et al., 1977; Eilers et al., 1993; Oller et al., 1994). Such studies have also found no reduction in volubility with a combination of prematurity and multilingual household. More recent research with infants of very low birthweight (<1000 g) has also revealed that, surprisingly, volubility appears to be at least as high as in full-term infants from 37 weeks gestational age through the middle of the first year after birth (Törölä et al., 2012a).

So according to present findings, infant volubility in the first year seems to be robust with regard to a variety of potential risks. The fact that hearing-impaired infants (and especially infants who are congenitally profoundly deaf) have not been found to show low volubility is particularly telling. It suggests that endogenous tendencies to vocalize run very deep in the human infant. It hints that parental influence on infant volubility is either very limited in the first year or that parental influence is primarily associated with visual (facial and gestural) and tactile interaction. The latter possibility fits with the fact that we *do* see low volubility in low SES (where we might expect overall interaction, including visual, tactile and vocal, to be reduced) and apparently in blindness, where visual interaction is absent.

## Conclusion

Our findings suggest that infants of one of our nearest genetic relatives, bonobos, do appear to produce protophone-like sounds, suggesting a commonality in language-like foundations with humans. Still, our report suggests human infant vocal communication contrasts sharply in frequency of occurrence with that of the bonobos we studied, and the difference calls for evolutionary explanation. Our approach represents an attempt to focus on the root of the vocal communicative differentiation of humans from the primate background, a differentiation that could not have begun with evolutionary changes immediately producing massive verbal lexicons, nor even with changes leading to small fully referential lexicons. Instead, the human power of language, as revealed in human infant development, depends on prior and more primitive capabilities, in particular the ability and inclination to vocalize spontaneously and frequently, without external stimulation, high arousal, or distress. All aspects of language depend upon the capability for spontaneous expression, and thus it seems no accident that human infants, from the first month of life, begin a playful, exploratory process that appears to be dramatically different from vocal activities of our closest relatives, forming a foundation for vocal interaction, which itself supplies further foundations without which language would be impossible (Oller et al., 2016). Both the human infant inclination to vocalize and the human parent inclination to engage that vocalization appear to have been naturally selected (Locke, 2006).

Our comparisons with ape infant vocalizations are, of course, only a beginning—they suggest the distinction between early human and early ape vocal capability is quantitatively large, but not absolute, as should be expected given our primate heritage. Additional direct cross-species quantification of vocal rates should help clarify roots of language and help to supplement research seeking those roots in gestural communication as well (Tomasello and Camaion, 1997; Hopkins and Leavens, 1998; Corballis, 2002; Call and Tomasello, 2007; Call, 2008; Iverson, 2010). The plentiful occurrence of human infant protophones reveals that raw material for vocal interaction is available to human caregivers from the first day of their infants’ lives, offering the potential to lay foundations for vocally-based bonding and indeed, for language.

## Author Contributions

DO developed the theoretical framework, served as primary writer of the paper, designed the human infant studies, and assisted in design of the bonobo study. UG developed the theoretical framework, contributed to the writing of the manuscript, and primarily designed and conducted the bonobo study. SI helped to develop the theoretical framework, contributed to the writing of the manuscript, helped to design the human infant studies, and primarily conducted one of the human infant studies. YJ contributed to the writing of the manuscript, helped to develop the theoretical framework, and helped in design and conducting of two of the human infant studies and in conducting the bonobo study. AW helped to develop the theoretical framework, contributed to the writing of the manuscript, and helped in design and conducting of two of the human infant studies. RD helped to develop the theoretical framework and contributed to the writing of the manuscript. JC helped to develop the theoretical framework, contributed to the writing of the manuscript, and assisted in designing and conducting the bonobo study.

## Conflict of Interest Statement

The authors declare that the research was conducted in the absence of any commercial or financial relationships that could be construed as a potential conflict of interest.

## Footnotes

1 For both laugh and cry/scream, the rates of occurrence were very low and quite variable across individuals, and as a result, no statistical comparison of the differences either between species or between laugh and cry/scream within species is warranted. The occurrence of these sounds in the bonobos was highly associated with circumstances that varied across individuals: Kasai produced the vast majority of the bonobo screams because he had a sister, who was sometimes present in the recordings, harassing him. Mobali produced the vast majority of the bonobo laughs because he had multiple adult females tickling him and doing rough and tumble play with him during many recordings.

2 There is reason to be cautious in judging the extent of active human infant involvement in vocal interaction especially at very young ages. We and others have speculated that shortly after birth, parents may be able to create a turn-taking frame for the infant involving little or no infant involvement in the interaction—the parent might merely wait for the infant to begin an endogenous vocal bout and then attempt to vocalize in alternation, thus selecting optimal times to entrain the infant in turn-taking. We speculate that parents may anticipate the onset of infant vocalizations (by observing respiratory patterns for example) and to curtail their own vocalizations, thus allowing the infant to speak uninterrupted. Such a tendency could create the illusory impression of active infant turn-taking.

## Supplementary Material

TABLE S1 Supplementary Methods.

DATA SHEET S1 Audio files for Supplementary Figures S1–S3.
