# Supplementary material for: Language Origins Viewed in Spontaneous and Interactive Vocal Rates of Human and Bonobo Infants
Source: Front Psychol. 2019 Apr 2;10:729. doi: 10.3389/fpsyg.2019.00729 (PMC6455048; doi:10.3389/fpsyg.2019.00729)
Supplement: TABLE S1 — Supplementary Methods. [file Table_1.docx]

***Supplementary Methods***

Language origins viewed in spontaneous and interactive vocal rates

of human and bonobo infants

**D. Kimbrough Oller, Ulrike Griebel, Suneeti Nathani Iyer, Yuna Jhang,**

**Anne S. Warlaumont, Rick Dale, Josep Call**

***Additional Methods***

***Recordings, equipment, and circumstances***

*Bonobo recordings:* The bonobo recordings were made with a Panasonic camcorder with a built-in microphone (sampling rate 40 kHz). Depending on the position and movement of the focal animals the camera was either mounted on a tripod or hand-held during recordings, which were monitored by the operator constantly. The infant was targeted if the mother and infant were separated, but usually both infant and mother were on camera.

The indoor facilities allowed recordings through the enclosure bars relatively close to the animals and where they were rarely more than 1-3 meters away from the camera/microphone and often were within one meter, because the animals were curious and could see the camera and its operator outside the enclosure. The Leipzig recordings were made in three adjacent facilities, 11m², 9m², and 5m² from left to right, with visual contact across them. The gates between them were sometimes left open so that the focal mother-infant pair was occasionally in one of the side facilities during recording, but usually they were in the middle one. The Memphis enclosure was much larger (~ 144 m^2^), and the focal pair was usually accompanied by two or three additional adult female bonobos, and occasionally by an adult male. The Memphis space was the primary indoor public viewing area for the bonobos, but there was a rear gated entrance for human caretakers where the camera operator could be seated and conduct the recordings through the bars of the gate.

The Leipzig facilities were much smaller spaces where the bonobos regularly engaged in various activities associated with the observational and experimental research conducted by the Max Planck Institute for Evolutionary Anthropology, so individual animals or mother-infant pairs were quite accustomed to entering and spending a period in those special indoor enclosures. In Leipzig, one or two other bonobos were often present in one of the two adjacent barred facilities, where visual, acoustic and tactile contact was possible with the focal animals. In the case of Yasa/Kasai, the adolescent sister, Fimi, was sometimes in the recording enclosure along with the mother and infant. This circumstance created some excitement during those sessions because Fimi often played rough and tumble with Kasai, and he often resisted, trying to get away and protesting vocally. In Leipzig, we recorded a small proportion of sessions where the mother-infant pair was not in visual contact with any other bonobos. Larger troupes of bonobos and other primates could often be heard outside these enclosures, sometimes in circumstances of high arousal and excitement. Sometimes the focal animals responded vocally to the outside activity, and these vocalizations of the focal animals were coded, although usually the functions and valences of these responses were uncertain.

With one exception, the maze task in Leipzig, we made no attempt to manipulate the circumstances of recording with the bonobos. Knowing human infants often vocalize when alone, we made a particular effort in Leipzig to give mothers something to do that might divert their attention from the infant and let him spend a period alone. The attempt was not successful, and we only pursued it for a small portion of the sessions. With Yasa (in four 15-minute sessions, 10% of the sessions for Yasa/Kasai) and Lexi (in a single session that only lasted 3 minutes because she seemed uninterested in the game) the maze task was administered, where the reward on each trial was a grape that had to be pushed by the bonobo through the maze with a stick until it could be reached by hand. The apparatus was presented through an opening in the recording enclosure. There was no tactile contact between humans and bonobos. Both Yasa and Lexi had learned the maze task before. Kasai witnessed the maze activity and instead of going off to play alone, as we had anticipated, he constantly interfered, and seemed to be excited by the game. Although he was unable to perform the task himself (he grabbed the stick and tried to get the grape himself several times), he excitedly harassed Yasa to hurry the process and give him the grape. This circumstance yielded quite a number of protophone-like infant vocalizations. Neither Yasa nor Lexi, during her short period of apparent boredom with the maze, showed any sign of excitement, even though Yasa had reason at least to be frustrated—Kasai often took Yasa’s stick away, dropping it outside the cage. He also found other ways to interfere, but Yasa remained patient and quiet.

Thus with three exceptions (during loud vocalizing from outside the enclosure in Leipzig for recordings of both Kasai and Yaro, during rough and tumble play in recordings of both Kasai and Mobali, and during the maze task with Kasai), which amounted to a small proportion of the bonobo recordings, the circumstances were calm. The relative calm was expected, since we recorded during periods not expected to involve high activity/high arousal, avoiding activities such as transfer of animals to other facilities, adding of new animals to the group, or new food supplies being distributed. During the recording sessions, for the most part, the bonobos spent their time grooming, resting, eating, and playing. The bonobo mothers and infants were typically very close together or in physical contact.

The recordings of Kiri/Mobali were conducted by the second author. During the recordings, there was always a zoo keeper present as well, a person familiar with the animals, and s/he expressed his/her opinion about who was vocalizing whenever it happened. This helped with coding since the environment was often noisy and multiple animals (at most 6) were involved. At the beginning, both the author/observer and the zoo keeper used hand signs visible on camera for indicating who vocalized, on the assumption that the animals might react vocally to speech, but it soon became clear that the animals did not react at all vocally to the spoken words, and from then on the coding indications were recorded orally. The second author also kept a written log of disagreements between her and the zoo keeper about which bonobo was vocalizing.

The recordings of the mother-infant pairs Lexi/Yaro and Yasa/Kasai were conducted by a student of the last author at the Leipzig zoo, and she indicated her opinions about who vocalized with hand signs, trying to give indications of every vocal event from the focal animals. The signing procedure was instituted because Kasai had reacted during the first recordings with vocalizations to human voices.

We recorded a total of 36 sessions (most about 30 minutes long) for Kiri/Mobali, 24 sessions (each about 15 minutes long) for Lexi/Yaro, and 38 sessions (each about 15 minutes long) for Yasa/Kasai. Due to considerable noise from cleaning equipment used intermittently nearby, some of the recorded data had to be excluded. Also we did not use or code periods when the animals were sleeping. We were left with 1770 minutes of recorded material for coded observation of vocal communication.

*Human recordings from the Memphis1 study:* The recording suite in Memphis consisted of two rooms, a control room and an adjacent one set up to resemble a child’s playroom (~10 m^2^) with 4 cameras mounted in the corners, offering good viewpoints to reliably code facial affect and functional valence in addition to vocal type, and to assess features of parent-infant interaction.^1^ The cameras were controlled for zoom and focus, and audio was monitored by an operator in the control room, who also selected, at each point of the recording, two of the four cameras, one focused on the infant’s face and one on the potential interaction, thus yielding video encompassing the parent, infant, and sometimes an additional person present.

Both parents and infants were fitted with wireless wearable microphones. For the infants, the placement in a vest was 7-12 cm from the infant’s mouth. A separate feed from the wireless microphones was used to provide a high fidelity audio signal from both microphones, digitized at 44-48 kHz, and each of the two channels of video/audio signal were synchronized to the high quality audio on the basis of a hand clap that was recorded in audio and video at the beginning of each recording. Thus the Memphis1 data comprise two-channels of video, along with two channels of high-sampling-rate audio, one from the parent’s microphone and one from the infant’s.

Recordings for the study were analyzed for three ages, 3, 6, and 11 months. The 54 recording sessions were typically 20 minutes in duration, 2 sessions per day. Sessions were regularly obtained in the low- to moderate-arousal circumstances of normal parental care,^2^ 1) where parent and infant interacted or 2) where infants were present in the recording room, but parents focused on responding verbally to the items of a questionnaire administered by a staff member. The primary published comparisons did not, however, focus on differences in recording circumstances, and the data across the circumstances have been collapsed for comparison of the Memphis1 data with the bonobo data in Figures 4 and 6 of the present paper. Also the Memphis1 study did not address volubility in seconds per minute; the calculations reported here of vocalizations in seconds/minute were developed from a reanalysis of the 1080 minutes of previously coded Memphis1 data. Additional details for methods of the Memphis1 study can be found in Oller et al. (2013).

*Human recordings from the Athens study:* The recording arrangement in Athens included three cameras, with microphones and sampling rates as in Memphis. The two-room physical layout was also similar to that in Memphis. The playroom was smaller, ~6 m^2^, and the cameras were mounted too high to usefully supply facial affect information. Consequently the study did not include affect or social function judgments. On the other hand, the data were reported at ages similar to those of the Memphis1 study, and the Athens study had the advantage of recordings in 3 circumstances, roughly randomized in order of occurrence across recording days, and corresponding to 3 different instructions to the parents: 1) In the no-adult-speech circumstance (NAS), the parent was instructed to read or conduct some other solitary activity while the infant was in the recording room with her for up to 10 minutes. Occasionally the parent was outside the recording room, and the infant was truly alone. Infants typically played by themselves during NAS, and the parent was silent. The other two circumstances were similar to those of the Memphis1 study: 2) the infant-directed-speech (IDS) circumstance, where the parent was instructed to play and verbally interact with the infant, and 3) the adult-directed-speech (ADS) circumstance, where the parent was in the recording room with the infant, but was engaged in an interview with an experimenter and was discouraged from seeking to engage the infant.

An attempt was made to record in each of the 3 circumstances for 10 minutes on each of the pre-selected recording days of the longitudinal research. This goal was adjusted based on infant state. The research yielded ~ 4800 minutes of codable recording distributed across the circumstances and ages. The data for analysis were selected without reference to how much vocalization they contained, because the study sought to compare the amount of vocalization in each setting. Additional details for the methods of the Athens study can be found in Iyer et al. (2016).

*Human recordings from the Memphis2 study:* The Memphis2 study was conducted in Memphis subsequent to the Memphis1 study. The recordings were made in infant homes with LENA recorders, 70-gram battery-powered devices (sampling rate = 16 kHz), worn in special clothing in a chest pocket (Oller et al., 2010; Zimmerman et al., 2009) with the microphone nominally 5-10 cm from the infant mouth.

The protocol for recording was standard for the LENA system (see Xu et al., 2014). Briefly, parents were provided with the appropriate clothing, were given instructions regarding how to charge the recorder overnight, how to turn it on and place it in the child’s clothing at wake up time, how to turn it off at bedtime and return it to the staff of the research project for processing. At bath times, parents were instructed to place the recorder near the bath area, as close to the infant as possible without letting it get wet.

The recordings lasted on average 13 hours, and were analyzed at 0, 1, 3, 6, 9, and 12 months. For the 0 month age (the first month after birth), we obtained a recording from 9 of the 12 infants, but at the remaining ages all 12 infants were represented. For human coding, we randomly selected 24 five-minute segments from each LENA recording. Thus there was a total of 8280 minutes of coded data. The Memphis2 study included no instructions to parents about how to interact with their infants on recording days.

***Coding***

*Coding software and procedure*: All coding (human and bonobo) was done in AACT (Action Analysis Coding and Training) by Delgado et al. (2010). Audio in AACT is displayed in a flexible acoustic analysis system, TF32 (Milenkovic, 2001). A cursor on the scrolling audio display (both spectrographic and waveform) indicates the location of audio with respect to frame accurate video at all times. For audio-only samples, the TF32 display was also available, scrolling during play and real-time coding. AACT allows coding in multiple fields (vocal type, facial affect, social function, etc.) and facilitates analysis of events occurring across fields with convenient utilities.

For the bonobo recordings, there was one main coder, author two, who also conducted the recordings of Kiri/Mobali, and thus was present in about half the recordings herself (about 15 hours). The coder was able through the AACT software to listen to utterances and simultaneously view them in video and TF32 (the acoustic display system) multiple times (repeat observation coding) before making a coding decision. The coding also required placement of cursors at the onset and offset of vocalizations in TF32, which then automatically provided duration information on each utterance. The coding category decisions along with the decisions about which animal had vocalized were based 1) on the information provided by the observer/camera operator during the recordings in the zoo, 2) on the signed or spoken indications of the operator or zoo keeper, which had been made at the time of the recordings, and 3) on repeated intensive passes of audio-video coding for the entire video footage of each session, where the coder went back and forth over the footage to check everything in the video with reference to the signed or spoken indications made during the recordings, instituting coding revisions when appropriate. Thus it can be said that all the coding was based on compromise decisions from the on-line judgments of camera operators and zoo personnel and repeat-listening/viewing of the recordings during the AACT coding. We emphasize the importance of the on-line observations because recording always reduces fidelity of the audio signal, the on-line observer having better access to the signal in terms of signal-to-noise ratio. In addition auditory localization is possible for an on-line observer, helping in discrimination of voices.

The primary coder for the bonobo studies reviewed and coded all the materials in AACT with at least four separate passes of each recording, viewing, listening, coding, and refining coding with multiple playbacks of events on all four passes. The coding created a record not only on the infant vocalizations along with functions/affect, but also a record on all the actions including vocalizations of any of the other animals (either in the same enclosure or outside) that might have influenced the infant. A detailed event log was maintained in the AACT coding files for anything that happened in the recordings before and after each infant vocalization in order to help supply justification for judgments of function and affect. All the data presented in Results in the main text are based on the primary coder’s final decisions.

There were multiple coders for all three human studies. In the case of the published studies we refer the reader to the original publications for details. The key difference between the two published studies and the Memphis2 study was that in the former, observers used repeat-observation coding (ROC), allowing them, when appropriate, to view and listen to each utterance multiple times. In the Memphis2 study, in contrast, we used real-time coding (RTC), with coders striking an appropriate key each time they heard an utterance meeting a specified criterion. With RTC much larger samples can be obtained, but durational information is sacrificed.

For the Memphis1 study, one complete pass of ROC on all 54 sessions addressed infant vocal type (with audio only), and each infant utterance was bounded by cursors, providing durational data for comparison with the bonobo study. A second complete pass in ROC was made for infant facial affect (with video only) during each period of a coded utterance, allowing assessment of emotional valence associated with each infant utterance. Additional passes for subsets of the 54 sessions were conducted in ROC with both audio and video for social functions of infant and parent utterances, allowing assessment of interaction and social intentions.

For the Athens study, audio and video was available at all times for ROC, which focused on infant protophones only. Durational data were obtained for each infant utterance.

For the Memphis2 study the coding team consisted of 9 normally-hearing female Masters students in Speech-Language-Pathology. Their coding was conducted in two separate RTC passes for each five-minute segment that had been selected from the all-day recordings, one to code the vocalizations of the infant (vocal type only) and one to code audible IDS (infant-directed speech) and ADS (adult-directed speech, which was defined to include speech directed towards anyone other than the infant wearing the LENA recorder) of all utterances spoken in the infant’s presence by adults and other children in that segment.

After coding the five minutes for infant vocal types, the coders responded to a number of questions, which included: 1) Did any other person talk to the baby? This could be the parent or another adult or child. 2) Did any other person talk to someone else? For example, two adults might have talked to each other, or a person might have talked on the phone. 3) Do you think the baby was alone in the room? And 4) Do you think the baby was asleep? The questions were answered on a 5-point scale, where 1 indicated never, 2 some of the time, 3 about half the time, 4 most of the time, and 5 the entire time. After responding to the first questionnaire, RTC of the segment for utterances of speakers other than the infant wearing the recorder was done, and after that, a second questionnaire was administered, including the following two questions, also invoking a 5-point scale: 1) Were any of the infant’s protophones used in vocal turn taking^4^ with another speaker? and 2) Were any of the infant’s protophones used purely for vocal play or vocal exploration?

*Coding categories, vocal:* During periods of observation, every non-vegetative utterance was coded except those occurring at such low intensity that they could scarcely be heard and thus would likely play no role in any communication. The same rule for elimination was used for both humans and bonobos.

Key observations made during coding included utterance durations (for seconds/minute assessments, Figures 4 and 5) and utterance counts (for utterance count data, Figure 6). A breath-group criterion (Lynch, Oller, Steffens, & Buder, 1995) was used to determine both bonobo and human utterance locations in time. Thus an utterance was defined as the period of phonation occurring on exhalation, with boundaries between utterances consisting of perceived inhalations or pauses. The breath-group criterion has become the standard for utterance determination in human vocal development research in part because it yields better coder agreement than methods based on predetermined timing criteria, and in part because vocal acts consist of modulations of the respiratory stream, with phonation occurring overwhelmingly on egress. Onset and offset of each infant bonobo scream and protophone-like vocalization were marked using the same criteria as in the case of the human vocalizations, yielding duration measures. Laughter in the bonobos was coded differently because it often consisted of rapid ingressive-egressive sequences that appear to be a standard form of bonobo (and chimpanzee) laughter. Separation into breath groups of this laughter was difficult; moreover, such separation was judged inappropriate given the seeming coherency of the ingressive-egressive units. For these ingressive-egressive laughs, we used a pause criterion to separate utterances, and have reported data on laughter only in terms of seconds/minute (Figure 4) on the grounds that the utterances/minute measures could not be obtained comparably for laughter and the other vocal types. Thus to maximize comparability, we have presented comparisons of the three vocal types (scream, laugh, protophone) as in Figure 4, only in terms of seconds/minute.

The time-domain (waveform) display supplemented by the spectrographic display was marked for onset of each utterance at the point at which the signal diverged discernibly from the noise background and was marked for offset at the point at which the signal disappeared into the noise background.

For the infant bonobo sounds, we initially coded at a detailed level, including numerous subcategories of infant bonobo sounds as suggested in existing literature (Bermejo & Omedes, 1999; Clay & Zuberbühler, 2011; de Waal, 1982). For comparison with human data, it was concluded, after detailed coding, that it was best to lump all the bonobo “laughs” together, all the “screams” together, and all the “other” non-vegetative vocalizations together. Vocalizations of the “other” group were judged by our coders to consist of graded versions of one or more types based on descriptors from prior literature (barks, hoos, peepyelps, grunts, etc.), and it was deemed unworkable for the present study to draw sharp distinctions among them—we treated these “other” sounds as protophone-like in our analyses, although it remained an empirical question to what extent they were functionally similar to protophones.

The human infant vocalizations in all the studies were coded for vocal type in such a way that they could be collapsed into the three groupings we settled on for comparison with the bonobo infant vocalizations. Human cry was treated as analogous to bonobo scream, human and bonobo laughter were deemed analogous, and all the human protophones (squeal, growl, vocant, raspberry, whisper, ingress, etc.) were treated as analogous to the “other” category of potentially protophone-like sounds of the bonobos.

*Coding categories, affective/functional and IDS/IDV:* We sought to compare affective valences and communicative functions accompanying vocalizations in both species. The coding of affect/function was easier with the human infants because facial affect and parental responses were usually easily interpretable by the human observers, as long as the participants were visible on camera. Assessing affect in the bonobo infants, on the other hand, was more difficult in part because it did not prove workable for the human observers to use facial affect as a basis for judgment except in cases of laughter. As a result, it was necessary to interpret apparent functional valences based on what was happening in and around each bonobo infant vocalization; for example, if the infant seemed frightened by nearby screams of other animals from outside the recording enclosure, was trying to escape from an annoyance, or seemed to be expressing frustration with trying to grasp something out of reach, the accompanying utterance was coded as negative. Also if the mother picked the infant up to get him out of trouble or helped him in another way, the coder took it to mean the mother had understood the infant’s complaint or plea. On occasion, a protophone-like utterance was coded as positive if it appeared the infant was particularly pleased with something, such as acquiring or eating a grape. In general other bonobo protophone-like utterances were coded as “don’t know”, meaning the coder did not feel confident that any affect or function judgment was justifiable.

Infant bonobo laughs were uniformly coded as positive and were always the result of physical play/tickling. In all cases where infant bonobo screams could be coded for affect (because the accompanying interaction had been visible), they were coded as negative, and the accompanying actions often included clear indications of infant upset, for example when Kasai went running to his mother trying to get away from his harassing older sister. Vocalizations other than screams that were coded as functionally negative included: Complaint vocalizations (for example, by Yaro when Lexi seemed to have hurt him during grooming by pulling out a hair); plea vocalizations (for example, when Kasai appeared to be hungry, and sought to breast feed); and vocal impatience expressions (when Mobali was climbing and pled for his mother to help him down from a high place, which she did). Thus the sequence of events observed in the recordings, including maternal reactions, was often used to supply contextual support for decisions about the valence and function of each vocalization.

The Memphis1 study had included additional coding for infant facial affect (positive, negative, neutral) occurring during vocalizations. The affect coding was based on smiling for positive and frowning for negative, with neutral corresponding to lack of smiling or frowning. This coding made possible comparison with the infant bonobo data on affect of vocal communications, although the bonobo infants’ affect was judged predominantly on the basis of situational factors (for example, tickle play was judged to yield positive affect, running away was judged to correspond to negative affect).

In addition, a subset of the Memphis1 data (12 interactive sessions from 9 infants across the first year) had been coded to indicate infant illocutionary force for each utterance. The term illocutionary force comes from Austin (1962), whose original usage was applied only to social functions of adult speech acts. The term has been adapted in our work (Oller & Griebel, 2008, 2014) to make it applicable to animal and human infant communication. Roughly illocutionary force is the function a communicative or potentially communicative act serves *in* the act of transmission. Thus, for example, complaint, exultation, conversational turn-taking, and vocal play are common human infant illocutionary forces, all of which are vocal expressions, but none of which necessarily implies the intentional transmission of information. These categories supply ways of characterizing the human infant’s role in vocal expression and interaction and to compare them with the roles played by vocal acts in bonobo infants without imputing semanticity to the infant bonobo behavior or shoe-horning the infant acts into more mature human categories such as phonemes or words. In particular the illocutionary-force coding of the subsample from the Memphis1 study supplied a way to estimate the extent to which human infants engaged in proto-conversation with the parents.

For example, if a human infant smiled and squealed looking directly at the mother, who applauded and smiled at the infant, the utterance would have been coded as exultation. If an infant produced a particular vocalization type repeatedly while showing no sign of positive or negative affect or of directing the vocalization to another person, it would have been coded as vocal play or vocal exploration, and the affect would have been coded as neutral.

Counts were also obtained for parent utterances directed toward the infant (IDS) in the Memphis1 subsample. In the case of the bonobos, Infant Directed Vocalizations (IDV) did not occur, yielding no vocal turn-taking events to monitor in the bonobos. The lack of IDV in the bonobos was determined by observing and coding every vocalization from any focal animal plus providing a commentary in AACT on the situation accompanying every infant vocalization, including whether the mother looked at the infant or vice versa. Sometimes the mothers responded to an infant plea physically, by picking the infant up or looking toward the infant, for example, but there was no case of a vocal response by a bonobo mother shortly after an infant vocalization. Furthermore, no maternal vocalization occurred while looking at an infant.

The great majority of vocalizations by the mothers were discernibly directed to another nearby adult bonobo in the recording enclosure or were interpreted as responses to loud events, including the loud vocalizations of the other bonobos outside. It should be emphasized that while the bonobo mothers did sometimes vocalize (63 instances were coded), they did so less than 1/10 as often as the bonobo infants.

Our criteria for judging IDV, if it had occurred, would have been based (as it was for IDS in humans) on two clear event types: 1) a caregiver producing a vocal response shortly after an infant vocalization, nominally within three seconds, especially if accompanied by gaze directed toward the infant or any other immediate caregiving physical interaction such as picking the infant up; or 2) a caregiver producing a vocalization accompanied by gaze directed to the infant or followed immediately by caregiving physical interaction with the infant. In humans, of course, IDS includes added features that can make it easy to identify (and can overrule the necessity to meet either of the other criteria); namely, the semantic content of the caregiver message may reveal that it is directed to the infant (e.g., you have to let mommy put you in the high chair now…”) or the message may be produced in baby register (parentese).

In the human case, we analyzed the subsample from the Memphis1 study where both infant and parent utterances had been coded. The judgment of IDS was made partly on the basis of the two criteria listed above. Caregivers usually looked at infants during IDS, their vocalizations in response to infant vocalizations typically occurred within 3 sec, and they often engaged in physical interaction immediately upon vocalization toward the infant. But usually none of these criteria needed to be invoked because the semantic content of the caregiver utterance made abundantly clear that the infant was the addressee (e.g., “it’s time to put you to sleep…”). In this way we were able to estimate the amount of IDS occurring during sessions designated for parent interaction with the infant along with rates of vocal turn-taking. If the caregiver responded vocally to the infant within ~ three seconds, the event was treated as both IDS and as turn-taking. Similarly, if the infant responded vocally to the caregiver within ~ three seconds, the event was treated as turn-taking.^4^

There was nothing we noticed that was unusual about the caregiver vocalizations in the human recordings—mostly they resembled IDS as portrayed in numerous articles addressing that topic in laboratories and homes ranging from moderately low to high SES in the USA, Europe, the Middle East, and Asia (Farran, Lee, Yoo, & Oller, 2016; Fernald, 1992; Glenn & Cunningham, 1983; Hirsh-Pasek et al., 2015; Kitamura, Thanavishuth, Burnhama, & Luksaneeyanawin, 2002; Paul, Chawarska, Fowler, Cicchetti, & Volkmar, 2007; Saint-Georges et al., 2013; Singh, Morgan, & Best, 2002; Thiessen, Hill, & Saffran, 2005). In general the adult IDS utterances were < 3 sec in duration and were often as short as .5 sec. The intonation patterns associated with baby register, one of the strongest indicators of IDS, have been described in the cited literature. Examples of IDS included instructions to infants (“take my finger”, “don’t hit me”), celebration (“that sound was wonderful”, “let’s do it again”), soothing (“oh, do you have a tummy ache?”), and projections about what might happen next (“let’s get you dressed for bed now”), and many cases where the caregiver produced versions of the baby’s sounds either as elicitations or as imitations, including all the protophone types illustrated in Figure 1.

*Coder preparation:* For the bonobo study, the second author evaluated the literature on bonobo vocalizations and established the coding protocol, which was ultimately collapsed for analysis into the categories scream, laugh, and other. A second individual (the fourth author) was trained by the second author to serve as an independent agreement coder on volubility of the infant bonobos. The first author was also instructed by the second author for bonobo infant vocalization coding. Example utterances used in the training were not drawn from among the utterances used in the agreement tests.

The first author provided gold-standard coding used in training for the Memphis1 and Memphis2 studies. All the coders had been trained in phonetic transcription during their program of university study. Training for the coding involved a two-hour introduction to infant vocalizations with both audio and audio-video examples presented primarily by the first author, whose work for decades has been focused on infant vocal development and the establishment of appropriate descriptive schemes for prelinguistic sounds (Oller, 1980). Thereafter, a series of practice tests were given during which each coder worked independently, coding infant vocal samples much like the ones that would be coded when the real data collection began. After three such rounds of coding and reviews of the results with the training staff and the first author the coders were certified to begin coding.

Similar procedures were utilized for training for the Athens study, with the third author serving to supply the gold-standard coding and face-to-face training. Details on training can be found in the original publications. In all the human studies, agreement coding was done by individuals who had been trained the same way.

*Coder agreement:* All the data used in the analyses on the bonobo data are those based on the primary coder’s work, including the compromises she settled on based on her own perceptions reviewing the recordings and the signed or written log indications that had been made by the on-line observers. She made multiple reviews of all the recordings, producing a voluminous record of careful coding, documenting not only the occurrence of vocalizations of all the focal animals, but also the circumstances preceding and accompanying them and their apparent affective expressions and functions, with ample cases deemed “don’t know” because the recordings seemed to offer no solid basis for a human to judge the function or affect of the vocalization event.

To test for coder agreement on the bonobo data, about 10 percent of the video material was also coded independently by the fourth author, who was experienced in the coding of human infant vocalizations, but naïve with respect to ape vocalizations, aside from the brief training provided by the primary coder, the second author. There were 11 recording sessions for the agreement coding (4 from Yasa/Kasai, 4 from Kiri/Mobali, and 3 from Lexi/Yaro). The correlation was very high between the coders on the number of protophone-like infant vocalizations indicated across the sessions (r = .98, n = 11). The primary coder indicated 177 protophone-like utterances had occurred in the coded agreement material, while the agreement coder indicated 210 in an unedited version of her coding, and 189 in an edited version that took account of a few clear errors of implementation of the coding instructions, as determined through an utterance-by-utterance review by the first and second authors after completion of the secondary coder’s work. The unedited version of the secondary coder’s work thus yielded a count that was 18% higher than that of the primary coder and the edited version 7% higher. This level of difference in volubility coding is not unusual with respect to differences we have seen across coders for volubility in human infants, and represents a magnitude >10 times smaller than the differences between human and bonobo infant volubility reported in Figures 4-6 of the main text based on the primary coder’s work. There were also 8 screams coded by the primary coder and 10 by the agreement coder in the samples.^3^

Given that this study represents the first attempt to quantify vocalization rates in human and non-human infants for direct comparison, it was deemed important also to compare the two versions of the coding on the agreement samples item by item. The first author reviewed each recording in AACT with the bounded and labeled utterances from both the primary and agreement coders shown on two rows below the acoustic display. As always in AACT, the corresponding video was available and was frame-accurately synchronized with the audio for playback in another window during this coding. The third row in the AACT/TF32 display was dedicated to the first author’s coding for all the agreement materials, a coding that was intended to reconcile, where possible, the differences between the other two versions of the coding (which were displayed on the first and second rows), thus developing a third estimate of the infant vocal rates in the bonobo agreement samples based on the first author’s judgments. The first author’s final count of protophone-like utterances for the 11 sessions was 169, about half a per cent lower than that of the primary coder.

The first author’s review of the coded agreement material and corresponding bonobo recordings in AACT yielded the following conclusions about differences of coding on individual protophone-like items: most disagreements were the product 1) of uncertainty about which animal had vocalized (the adult bonobos sometimes produced vocalizations that were hard to distinguish from vocalizations of the infants) or 2) of differences regarding inclusion by coders of very low intensity or short duration vocalizations. The same disagreement types are common in coding of human infant vocalizations.

A potentially important methodological finding of this work is that counting bonobo infant protophone-like utterances in recordings obtained in circumstances such as these (low-to-moderate arousal, few animals present, and cameras/microphones only a few meters away from the animals) is apparently very workable. This is heartening, since the recording circumstance we faced with the bonobos was hardly optimal. Unlike the case of recordings we make with human infants, recording equipment with microphones cannot currently be placed on bonobos to minimize mouth-to-microphone distance. It is also true that the voices of infant and mature bonobos were often surprisingly difficult to distinguish. Yet the agreement level for protophone-like utterance counts of the bonobo infants were not very different from those we have obtained in research on human infant protophones.

The first author also conducted two independent passes of the recordings in AACT to code affect accompanying bonobo infant protophone-like utterances. He used a stricter criterion for judgment of negativity in the one case than in the other. He judged 111 infant bonobo utterances (60% of the protophone-like utterances he had coded in the vocal type dimension) as negative with the strict affect criterion and 135 (73%) as negative with the less strict criterion. The primary coder had judged 97 (49% of the infant utterances she coded in the agreement samples) to express negative affect. Thus the agreement data suggest the primary coder (who produced all the final data used in the analysis) had adhered to a conservative criterion in judging negativity, using the “don’t know” option for coding of affect liberally.

Thus for both the primary coder and the first author, a large proportion of the protophone-like utterances were deemed to be not determinable for affect. Further, the data suggested clearly that the method of affect coding in the bonobos was not as reliable as the method of coding vocalization types in these recordings. An important challenge for any future work of this sort will be to seek better ways of assessing affect and function of the communicative actions of non-human primates.

Agreement data for coding in the Memphis1 and Athens studies is provided in the cited publications (Iyer et al., 2016; Oller et al., 2013). To summarize regarding the most common infant vocal types, agreement across blind coding of randomly selected recording segments resulted in counts of protophones and cries that correlated at r ~ 0.9 across sessions, with average discrepancies across coders of ~ 10% of the mean counts for sessions.

To assess reliability of the RTC of all-day recordings of human infants, a PhD student with considerable experience using the system coded 408 five-minute segments distributed approximately evenly across infants and samples. The correlation for number of protophones between the 408 agreement samples and the standard (coding team) coders’ samples was 0.90, and for cries the correlation was 0.88. Laughs were so infrequent as to make agreement assessment in the randomly selected samples unworkable. The correlation for the questionnaire items were as follows: for the 5-point scale on the IDS question, 0.82, for the adult-to-adult talk question, 0.79, for the baby sleep question, 0.70, and for the baby alone question, 0.69.

The coding team and reliability coders differed by < 10% of the mean for counts of the combined category of protophones plus cries across the 408 samples that were coded by both, an average discrepancy of 0.34 utterances per minute across the ages. For the questionnaire items, the grand mean discrepancies as a proportion of the range of possible values (1-5) for the four questions were: 0.05 (talk to baby, IDS), 0.08 (adult-to-adult talk), 0.08 (baby asleep), and 0.03 (baby alone).

The coding of affect into three categories (positive, negative, neutral) for the human protophones yielded a kappa coefficient of 0.77 for 9 intercoder agreement samples judged by two independent coders from the Memphis1 study as reported in the Supporting Information Appendix to Oller et al. (2013).

**Footnotes**

^1^The cameras were mounted on poles and could be slid up and down to offer optimized viewpoints, depending on infant body position (for example, a seated infant was best viewed with a low camera, and a supine infant with a high camera).

^2^In the Memphis1 study, if an infant persistently cried during a recording session, or if s/he fell asleep, the session was terminated to be reinitiated either after a rest period or on another day. Recording sessions where infants vocalized at extremely low rates (usually because of drowsiness) were not included in the analyses of the Memphis1 study. Similarly, analyses of the bonobo data excluded periods of infants sleeping.

^3^For both laugh and cry/scream, the rates of occurrence were very low and quite variable across individuals, and as a result no statistical comparison of the differences either between species or between laugh and cry/scream within species is warranted. The occurrence of these sounds in the bonobos was highly associated with circumstances that varied across individuals: Kasai produced the vast majority of the bonobo screams because he had a harassing sister. Mobali produced the vast majority of the bonobo laughs because he had multiple adult females tickling him and doing rough and tumble play with him during many recordings.

^4^There is reason to be cautious in judging the extent of active infant involvement in vocal interaction especially at very young ages. We have speculated that shortly after birth, parents may be able to create a turn-taking frame for the infant involving little or no infant involvement in the interaction—the parent might merely wait for the infant to begin an endogenous vocal bout and then attempt to vocalize in alternation, thus selecting optimal times to entrain the infant in turn-taking. We speculate that parents may be able to anticipate the onset of infant vocalizations (by observing respiratory patterns for example) and to curtail their own vocalizations, thus allowing the infant to speak uninterrupted. Such a tendency could create the illusory impression of active infant turn-taking.

**References**

Bermejo, M., & Omedes, A. (1999). Preliminary vocal repertoire and vocal communication of wild bonobos (Pan paniscus) at Lilungu (Democratic Republic of Congo). *Folia Primatologica, 70*, 328-357.

Clay, Z., & Zuberbühler, K. (2011). Bonobos Extract Meaning from Call Sequences. *PLoS One, 6*(4), 1-10.

de Waal, F. (1982). The Communicative Repertoire of Captive Bonobos (Pan paniscus), Compared to That of Chimpanzees. *Behaviour, 106*(3/4), 183-251.

Delgado, R. E., Buder, E. H., & Oller, D. K. (2010). AACT (Action Analysis Coding and Training). Miami, FL: Intelligent Hearing Systems.

Farran, L. K., Lee, C.-C., Yoo, H., & Oller, D. K. (2016). Cross-Cultural Register Differences in Infant-Directed Speech: An Initial Study. *PLoS One, 11*(3), e0151518. https://doi.org/10.1371/journal.pone.0151518

Fernald, A. (1992). Meaningful melodies in mothers' speech to infants. In H. Papoušek, U. Jürgens, & M. Papoušek (Eds.), *Nonverbal vocal communication: Comparative and developmental approaches. Studies in emotion and social interaction.* (pp. 262-282). New York: Cambridge University Press.

Glenn, S. M., & Cunningham, C. C. (1983). What do babies listen to most? A developmental study of auditory preferences in nonhandicapped infants and infants with Down's syndrome. *Development Psychology, 19*, 332-337.

Hirsh-Pasek, K., Adamson, L. B., Bakeman, R., Owen, M. T., Golinkoff, R. M., Pace, A., . . . Suma, K. (2015). The contribution of early communication quality to low-income children’s language success. *Psychological Science, 26*, 1071–1083. doi: 10.1177/0956797615581493

Iyer, S. N., Denson, H., Lazar, N., & Oller, D. K. (2016). Volubility of the human infant: Effects of parental interaction (or lack of it). *Clinical Linguistic & Phonetics*, 1-19. doi: 10.3109/02699206.2016.1147082.

Kitamura, C., Thanavishuth, Burnhama, D., & Luksaneeyanawin, S. (2002). Universality and specificity in infant-directed speech: Pitch modifications as a function of infant age and sex in a tonal and non-tonal language. *Infant Behavior & Development, 24*, 372–392.

Lynch, M. P., Oller, D. K., Steffens, M. L., & Buder, E. H. (1995). Phrasing in prelinguistic vocalizations. *Developmental Psychobiology, 28*, 3-23.

Milenkovic, P. (2001). TF32 [Computer software]. Madison, WI: University of Wisconsin- Madison.

Oller, D. K. (1980). The emergence of the sounds of speech in infancy. In G. Yeni-Komshian, J. Kavanagh, & C. Ferguson (Eds.), *Child phonology, Vol 1: Production* (pp. 93-112). New York: Academic Press.

Oller, D. K., Buder, E. H., Ramsdell, H. L., Warlaumont, A. S., Chorna, L., & Bakeman, R. (2013). Functional flexibility of infant vocalization and the emergence of language. *Proceedings of the National Academy of Sciences, 110*(16), 6318-6632. doi:10.1073/pnas.1300337110

Oller, D. K., & Griebel, U. (2008). Complexity and flexibility in infant vocal development and the earliest steps in the evolution of language. In D. K. Oller & U. Griebel (Eds.), *Evolution of Communicative Flexibility: Complexity, Creativity and Adaptability in Human and Animal Communication* (pp. 141-168). Cambridge, MA: MIT Press.

Oller, D. K., & Griebel, U. (2014). On Quantitative Comparative Research in Communication and Language Evolution. *Biological Theory, 9*(2). doi:10.1007/s13752-014-0186-7

Oller, D. K., Niyogi, P., Gray, S., Richards, J. A., Gilkerson, J., Xu, D., . . . Warren, S. F. (2010). Automated Vocal Analysis of Naturalistic Recordings from Children with Autism, Language Delay and Typical Development. *Proceedings of the National Academy of Sciences, 107*(30), 13354-13359.

Paul, R., Chawarska, K., Fowler, C., Cicchetti, D., & Volkmar, F. (2007). “Listen my children and you shall hear”: auditory preferences in toddlers with autism spectrum disorders. *Journal of Speech, Language, and Hearing Research, 50*, 1350-1364.

Saint-Georges, C., Chetouani, M., Cassel, R., Apicella, F., Mahdhaoui, A., Muratori, F., . . . Cohen, D. (2013). Motherese in Interaction: At the Cross-Road of Emotion and Cognition? (A Systematic Review). *PLoS One, 8*(10), e78103.

Singh, L., Morgan, J. L., & Best, C. T. (2002). Infants' listening preferences: Baby talk or happy talk? *Infancy, 3*(3), 365-394.

Thiessen, E. D., Hill, E. A., & Saffran, J. R. (2005). Infant-Directed Speech Facilitates Word Segmentation. *Infancy, 7*(1), 53-71.

Zimmerman, F., Gilkerson, J., Richards, J., Christakis, D., Xu, D., Gray, S., & Yapanel, U. (2009). Teaching By Listening: The Importance of Adult-Child Conversations to Language Development. *Pediatrics, 124*, 342-349.
